# Targeted proteomic approach in prostatic tissue: a panel of potential biomarkers for cancer detection

**DOI:** 10.18632/oncoscience.313

**Published:** 2016-07-08

**Authors:** Donatella Aiello, Francesca Casadonte, Rosa Terracciano, Rocco Damiano, Rocco Savino, Giovanni Sindona, Anna Napoli

**Affiliations:** ^1^ Department of Chemistry and Chemical Technologies, University of Calabria, Italy; ^2^ Department of Health Sciences, Magna Græcia University of Catanzaro, Catanzaro, Italy

**Keywords:** PCa tissue, biomarker, metabolic pathway, bodily fluids, proteome

## Abstract

Prostate cancer (PCa) is the sixth highest causes of cancer-related deaths in men. The molecular events underlying its behavior and evolution are not completely understood. Prostate-specific antigen (PSA) is the only approved Food and Drug Administration biomarker. A panel of ten stage-specific tumoral and adjacent non tumoral tissues from patients affected by PCa (Gleason score 6, 3+3; PSA 10 ÷19 ng/ml) was investigated by MS-based proteomics approach. The proposed method was based on identifying the base-soluble proteins from tissue, established an efficient study, which lead to a deeper molecular perspective understanding of the PCa. A total of 164 proteins were found and 132 of these were evaluated differentially expressed in tumoral tissues. The Ingenuity Pathway Analysis (IPA) showed that among all dataset obtained, 105 molecules were involved in epithelial neoplasia with a p-value of 3.62E-05, whereas, only 11 molecules detected were ascribed to sentinel tissue and bodily fluids.

## INTRODUCTION

Prostate cancer (PCa) is the second most common cancer diagnosis worldwide and the sixth highest causes of cancer-related deaths in men [1]. Genetic, environmental factor, age, hormonal imbalance and diet denote the risk factor for PCa development. The detection and diagnosis of PCa are carried out by the measurement of serum prostate-specific antigen (PSA) level, digital rectal exam and histological inspection of prostate tissue biopsy [2]. PSA is the only biomarker approved by Food and Drug Administration (FDA). This test is useful for early diagnosis reducing the mortality, whereas the low sensitivity and specificity lead to overdiagnosis and overtreatment [3]. The misdiagnosis of PCa results in an non-predicable and aggressive treatment which may initiate a series of molecular events, which are not well understood. Therefore, to improve the diagnosis specificity and the clinical management the identification of additional biomarkers is desirable. DNA microarrays [4] can be used to measure PCa by providing the ability to compare changes in gene expression in the developing of PCa; however, they do not allow measurements of the protein levels. Proteomics represent a promising approach for the discovery and identification of specific molecules or set of proteins that are characteristics of a pathologic state [5]. Proteomics analysis of specific tissue can elucidate the mechanism of cells transformation from normal to cancerous status and provide a specific set of proteins to differentiate aggressive or indolent cancer forms. To date, analyses of protein levels in cancer have been performed by either using two-dimensional (2D) PAGE and/or surface enhanced laser desorption/ionization (SELDI) mass spectrometry [6]. Several studies describe the use of isobaric-tags for relative and ab significant upregulation of proteins, alpha-1-antitrypsin, which is a well-known as biomarker for inflammation and α-methylacyl CoA racemase. Sun et al. [9] analysed prostate tissue from BPH, PCa and BPH with local prostatic intraepithelial neoplasm and identified periostin as a potential biomarker for prostate cancer. It is well known that carcinogenesis produces in biological fluids cancer molecular specific biomarkers. These biomarkers result from complex biological phenomena which are supported by a rich network of different cells such as fibroblasts, endothelial cells, immune and inflammatory cells, extra-cellular matrix and proteins produced by the malignant microenvironment [10]. In an effort to identify a set of specific molecules which are associated with cancer development, in prostate tissues and biological fluids, we have developed an alternative method based on the extraction of hydro-soluble tissue proteins followed by protein fractionation compatible with mass-spectrometry analysis. In addition, tumoral and histological adjacent benign tissues of prostate from patients with elevated PSA value and Gleason Grade were selected as case studies to identify and quantify potential prostate tumor markers [11, 12]. A selective solubilization procedure was adopted to extract hydrosoluble basic proteins from prostate tissue. Then, protein depletion was performed to remove interfering highly abundant proteins; this removal unmasks low abundance proteins of interest for further investigation. The proteins were then subjected to solution phase trypsin proteolysis followed by iTRAQ-labelling and finally analysed by LC-MALDI MS/MS. Using this approach we found 164 proteins. 132 proteins were differentially expressed, 11 proteins were expressed in bodily fluids and these can be used as potential cancer biomarkers for PCa diagnosis.

## RESULTS

An alternative and rapid protocol has been developed for selective protein solubilization [13–15] from prostate tissue, followed by iTRAQ labelling, HPLC fractionation and MALDI MS/MS analysis to identify a set of specific markers for PCa diagnosis. The procedure was optimized on the swine prostate tissue which is considered the best classic biomedical model for human disease [16]. High abundant proteins were depleted by two different commercial columns using alternative MS-compatible buffers and the resulting fractions were visualized by SDS-PAGE in order to check the efficiency of the planned procedure (Figure S1). Multiple Affinity removal spin cartridge was chosen as the optimal depletion device because it is able to carry out several runs with no memory effect.

**Figure 1:**
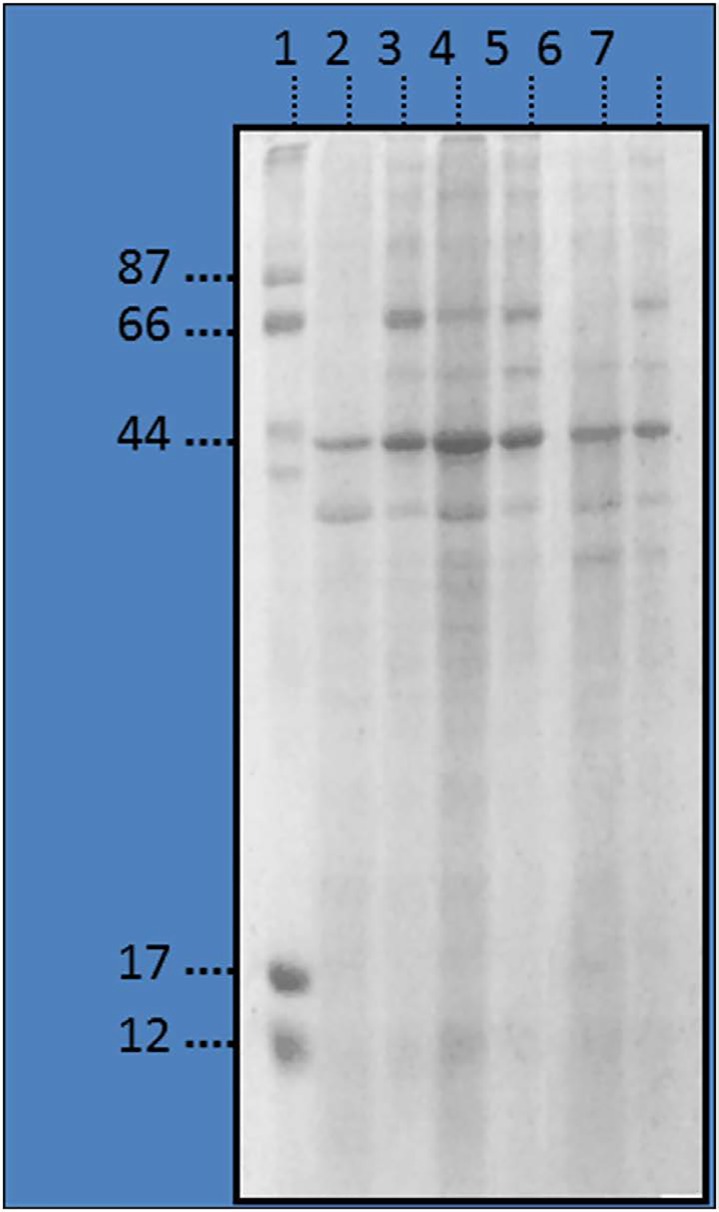
Electrophoresis profile of prostate human tissue Lanes: 1. Marker. 2-3: Depleted and whole fractions from human tumoral (T) prostate tissue from patient A. 4-5: Depleted and whole fractions from human tumoral (T) prostate tissue from patient B. 6-7: Depleted and whole fractions from human non tumoral (NT) prostate tissue from patient B.

The optimized sample preparation procedure was used for human prostate tissue. SDS-PAGE and MALDITOF MS profiles of the resulting fractions are reported in Figure 1 and Figure SI2, respectively. The major proteins solute quantitation (iTRAQ) for the investigation of prostate tissue in order to identify potential markers for cancer diagnosis, prognosis or treatment. [7] Garbis et al. [8] analyzed prostate tissue from patients with benign prostatic hyperplasia (BPH) and with prostate cancer thought iTRAQ labelling. Sixty five differentially expressed proteins have been previously described as specific marker for prostate cancer cells. These were identified as: prostaglandin E synthase resulting from are removed providing access to the next level of protein (hLA) as shown in Figure 1. SDS-PAGE shows different protein profiling of whole protein extracts (Figure 1, lines 3, 5 and 7) and hLA fractions (Figure 1, lines 3, 4 and 6). The experimental conditions for i-TRAQ quantitative analysis were modified (see experimental section). A total of 164 proteins were identified and 132 were considered differentially expressed between T and NT prostate tissue, with ion ratio of either ≥ 2 or ≤ 0.5 at p-value less than 0.05 for statistical significance (Table 1). Proteins were identified and quantified with no minus of three labelled peptides. The experiments were performed in triplicate and all peptide sequences are reported in Table SI2 and SI3 (Supporting Information).

**Table 1:** Identified proteins from tumoral and non tumoral prostate tissue by MS/MS data processing^a^

	Accession Number^b^	Gene Name^b^	Protein Name^b^	IP^c^	MW(Da)^c^	Location^b^	Biological Processes and Molecular Function^b^	Quantification 117:115^a^
**1.**	**P63104**	**YWHAZ**	14-3-3 protein zeta/delta*	473	27745	cytoplasm	adapter protein implicated in the regulation of signaling pathways negative regulation of apoptotic process	380
**2.**	**Q9P2A4**	**ABI3**	**ABI gene family member 3**	499	39035	cytoplasm	regulation of cell migration	180
**3.**	**P68032**	**ACTC1**	Actin alpha cardiac muscle 1*	523	42019	cytoskeleton	cell structure and motility	227
**4.**	**P25054**	**APC**	Adenomatous polyposis coli protein*	793	311646	cytoplasm	signal transduction oncogenesis beta-catenin binding, protein kinase regulator activity	292
**5.**	**O95996**	**APC2**	**Adenomatous polyposis coli protein 2**	908	243949	cytoplasm and cell membrane	promotes rapid degradation of CTNNB1 and may function as a tumor suppressor May function in Wnt signaling	358
**6.**	**Q08462**	**ADCY2**	Adenylate cyclase*	840	123603	Citoplasm/membrane	membrane-bound and Adenylate cyclase activity	320
**7.**	**P51825**	**AFF1**	AF4/FMR2 family member 1*	926	131422	nucleus	oncogene transcription factor-	237
**8.**	**P10696**	**ALPPL2**	**Alkaline phosphatase placental-like**	590	57377	membrane	hydrolase with biological process unclassified	145
**9.**	**Q99490**	**AGAP2**	Arf-GAP with GTPase ANK repeat and PH domain-containing protein 2*	991	124674	cytoplasm and nucleus	protein transport oncogenic overexpressed in cancer cells prevents apoptosis and promotes cancer cell invasion	302
**10.**	**Q8TF01**	**PNSIR**	Arginine/serine-rich protein PNISR*	1002	92577	Nucleus cytoplasm	transcription system	159
**11.**	**O14525**	**ASTN1**	**Astrotactin 1 (Fragment)** *	509	144913	membrane	cell adhesion	255
**12.**	**Q4LE39**	**ARID4B**	AT-rich interactive domain-containing protein 4B*	504	147809	Nucleus and cytoplasm	transcriptional repressor	217
**13.**	**O75815**	**BCAR3**	**Breast cancer anti estrogen resistance protein 3**	819	92566	intracellular	guanine nucleotide responsive factor signal trasduction	721
**14.**	**Q9UIF8**	**BAZ2B**	Bromodomain adjacent to zinc finger domain protein 2B*	613	240459	nucleus	transcriptional regulation	164
**15.**	**Q9NYQ7**	**CELSR3**	**Cadherin EGF LAG seven-pass G-type receptor 3**	623	358185	cell membrane	cell signaling receptor	295
**16.**	**O15484**	**CAPN5**	Calpain-5*	757	73169	cell surface	hydrolase involved in protein metabolism and modification	116
**17.**	**Q66K79**	**CPZ**	**Carboxypeptidase Z precursor** *	822	73655	Secreted) extracellular space	metalloprotease biological process unclassified	389
**18.**	**P35222**	**CTNNB1**	Catenin beta-1*	553	85497	cytoplasm nucleus cell membrane	cell adhesion transcription regulation and oncogenesis	210
**19.**	**Q96P48**	**ARAP1**	**Centaurin-delta-2**	586	162192	Golgi apparatus membrane cytoplasm	GTPase activation	578
**20.**	**Q9HC77**	**CENPJ**	Centromere protein J*	623	153000	Cytoplasm cytoskeleton	plays an important role in cell division and centrosome function	159
**21.**	**O14647**	**CHD2**	**Chromodomain-helicase-DNA-binding protein 2 (CHD-2)** *	822	211344	nucleus	transcription regulation DNA-binding helicase	149
**22.**	**Q8TD26**	**CDH6**	Chromodomain-helicase-DNA-binding protein 6*	590	305412	nucleus	transcription regulator	353
**23.**	**Q02388**	**COL7A1**	**Collagen alpha 1(VII)** *	595	295220	secreted extracellular space	extracellular matrix structural constituent	207
**24.**	**P08123**	**COL1A2**	Collagen alpha 2(I) chain* ^**^*	908	129314	secreted extracellular space	extracellular matrix structural constituent	319
**25.**	**P08572**	**COL4A2**	Collagen alpha 2(IV) chain*	885	167553	secreted extracellular space	extracellular matrix structural constituent	312
**26.**	**P12277**	**CKB**	Creatine kinase B-type*	535	42644	cytoplasm	central role in energy transduction in tissues	449
**27.**	**Q9P0U4**	**CXXC1**	**CXXC-type zinc finger protein 1**	861	75712	nucleus	transcription regulation	110
**28.**	**Q9NZJ0**	**DTL**	**Denticleless protein homolog**	911	79468	Nucleus and cytoplasm	cell cycle control DNA damage response and translation DNA synthesis	314
**29.**	**P17661**	**DES**	**Desmin** *	521	53536	cytoplasm	cytoskeletal protein binding muscle protein	378
**30.**	**Q08554**	**DSC1**	**Desmocollin 1A/1B precursor**	525	99987	cell membrane	cell adhesion-mediated signaling	255
**31.**	**Q14117**	**DPYS**	**Dihydropyrimidinase** *	681	56630	cytoplasm	nucleoside nucleotide and nucleic acid metabolism	630
**32.**	**Q9Y485**	**DMXL1**	**DmX-like 1 protein** *	591	337839	extracellular space	unknown	614
**33.**	**Q9NPF5**	**DMAP1**	**DNA methyltransferase 1-associated protein 1**	951	52993	Cytoplasm and nucleus	transcription repression and activation	227
**34.**	**Q92878**	**RAD50**	DNA repair protein RAD50*	647	153892	nucleus	hydrolase	401
**35.**	**O60870**	**KIN**	**DNA/RNA-binding protein KIN17**	907	45374	nucleus and cytoplasm	involved in DNA replication and the cellular response to DNA damage	050
**36.**	**Q8TD84**	**DSCAML1**	Down syndrome cell adhesion molecule-like protein 1*	843	224463	cell membrane	cell adhesion and neurogenesis	380
**37.**	**Q96DT5**	**DNAH11**	Dynein heavy chain 11 axonemal*	603	521043	cytoplasm	force generating protein of respiratory cilia produces force towards the minus ends of microtubules and has ATPase activity	492
**38.**	**Q8WXX0**	**DNAH7**	**Dynein heavy chain 7 axonemal** *	570	461159	cytoplasm cytoskeleton microtubule	force generating protein of respiratory cilia produces force towards the minus ends of microtubules and has ATPase activity	443
**39.**	**Q03001**	**DST**	Dystonin*	514	860662	cytoplasm and cytoskeleton	integrator of intermediate filaments involved in actin and microtubule cytoskeleton networks	428
**40.**	**P14625**	**HSP90B1**	Endoplasmin*	476	92469	endoplasmic reticulum	molecular chaperone	391
**41.**	**Q96J88**	**EPSTI1**	**epithelial stromal interaction protein 1**	990	36793	unspecified	unknown	265
**42.**	**Q8TAM0**	**GPR62**	G protein-coupled receptor 62*	1099	37619	Cell membrane	G-protein coupled receptor	301
**43.**	**O94808**	**GFPT2**	**Glucosamine-fructose-6-phosphate aminotransferase [isomerizing] 2**	703	76931	cytosol	aminotransferase	224
**44.**	**Q6PCE3**	**PGM2L1**	**Glucose 16-bisphoshate syntase**	681	70442	cytosol	glucose metabolism isomerase and transferase	303
**45.**	**P30711**	**GSTT1**	Glutathione S-transferase theta 1*	701	27335	cytoplasm	glutathione transferase activity	173
**46.**	**Q9NU53**	**GINM1**	**Glycoprotein integral membrane protein 1**	481	36840	membrane	unspecified	127
**47.**	**Q14789**	**GOLGB1**	Golgin subfamily B member 1*	496	376019	Golgi apparatus and membrane	unknown	050
**48.**	**Q99062**	**CSF3R**	Granulocyte colony stimulating factor receptor*	576	92156	cell membrane	receptor	354
**49.**	**Q03113**	**GNA12**	Guanine nucleotide- binding protein subunit alpha-12*	984	44279	membrane	modulators or transducers in various trans-membrane signaling systems controller of cell migration through the TOR signaling cascade	379
**50.**	**Q96LI6**	**HSFY1**	**Heat shock transcription factor Y-linked**	668	45107	nucleus cytoplasm	transcription regulation	110
**51.**	**P69905**	**HBA1**	**Hemoglobin subunit alpha**	872	15258	cytosol	oxygen transporter	497
**52.**	**P68871**	**HBB**	**Hemoglobin subunit Beta**	674	15998	cytosol	oxygen transporter	271
**53.**	**P09105**	**HBQ1**	**Hemoglobin subunit theta-1**	709	15508	cytosol	oxygen transporter	288
**54.**	**Q8TEK3**	**DOT1L**	**Histone-lysine N-methyltransferase H3 lysine-79 specific** *	939	184853	nucleus	chromatin regulator	304
**55.**	**P17482**	**HOXB9**	Homeobox protein Hox-B9*	901	28059	nucleus	sequence-specific transcription factor	249
**56.**	**Q9HAS2**	**HIPK3**	Homeodomain-interacting protein kinase 3*	716	133743	cytoplasm and nucleus	serine/threonine-protein kinase involved in transcription regulation apoptosis and steroidogenic	907
**57.**	**P42858**	**HTT**	**Huntingtin**	581	347603	cytoplasm and nucleus	may play a role in microtubule-mediated transport or vesicle function Protein binding	388
**58.**	**Q9Y4L1**	**HYOU1**	Hypoxia up-regulated protein 1*	516	111335	nucleus	protein metabolism and modification	229
**59.**	**P23677**	**ITPKA**	**Inositol 145-trisphosphate 3-kinase A**	759	51009	cytosol	kinase	299
**60.**	**O15503**	**INSIG1**	Insulin-induced protein 1*	908	29987	endoplasmic reticulum membrane	protein binding may play a role in growth and differentiation of tissues involved in metabolic control and has a regulatory role during G0/G1 transition of cell growth	098
**61.**	**P24593**	**IGFBP5**	Insulin-like growth factor binding protein 5 precursor*	858	30570	secreted	signal transduction and cellular protein metabolic process	306
**62.**	**Q9BR39**	**JPH2**	**Junctophilin 2**	882	74222	cell membrane	contribute to the formation of junctional membrane complexes and to the construction of skeletal muscle triad junctions	477
**63.**	**Q01546**	**KRT76**	**Keratin type II cytoskeletal 2 oral**	838	65841	cytoskeletal	cell structure and motility	293
**64.**	**Q96L93**	**KIF16B**	Kinesin-like protein KIF-16B*	586	152011	cytoplasm	motor protein involved in endosome transport and receptor recycling and degradation	456
**65.**	**Q8N4N8**	**KIF2B**	Kinesin-like protein KIF2B*	889	76254	cytoplasm	motor protein required for spindle assembly and chromosome movement	470
**66.**	**Q32MZ4**	**LRRFIP1**	Leucine-rich repeat flightless-interacting protein 1*	459	89253	nucleus and cytoplasm	transcriptional repressor	381
**67.**	**Q9UNZ5**	**C19orf53**	**Leydig cell tumor 10 kDa protein homolog**	1155	10577	nucleus	potential role in hyper-calcemia of malignancy	449
**68.**	**Q9H2C1**	**LHX5**	**LIM/homeobox protein Lhx5**	787	44406	nucleus	transcription regulation	412
**69.**	**O75334**	**PPFIA2**	Liprin-alpha2*	580	143291	cytoplasm and cell surface	protein binding	261
**70.**	**Q9NZR2**	**LRP1B**	Low-density lipoprotein receptor-related protein 1B*	509	515498	membrane	cell surface proteins involved in endocytosis	261
**71.**	**Q9H239**	**MMP28**	**Matrix metalloproteinase-28**	970	58939	Secreted/extracellular space	could play a role in tissues homeostasis and repair	282
**72.**	**Q9NR99**	**MXRA5**	Matrix-remodeling-associated protein 5*	857	312150	secreted	unknown but it is overexpressed in centenarians	332
**73.**	**Q96JG8**	**MAGED4**	**Melanoma-associated antigen D4**	634	81378	unspecified	tumor antigen	214
**74.**	**Q8NFU7**	**TET1**	Methylcytosine dioxygenase TET1*	853	235309	nucleus	transcription regulation activator and regulator	067
**75.**	**P11137**	**MAP2**	Microtubule-associated protein 2*	482	199526	cytoplasm	may stabilize the microtubules against depolymerization	272
**76.**	**Q9NU22**	**MDN1**	Midasin*	546	632820	nucleus	nuclear chaperone required for maturation and nuclear export of pre-60S ribosome subunits	449
**77.**	**P08235**	**NR3C2**	**Mineralocorticoid receptor (MR)** *	722	107067	cytoplasm nucleus endoplasmic reticulum membrane	nuclear hormone receptor and transcription factor	262
**78.**	**O60336**	**MAPKBP1**	**Mitogen-activated protein kinase-binding protein 1**	631	163818	unknown	involved in JNK signaling pathway	500
**79.**	**Q8WV50**	**BUB1B**	Mitotic checkpoint serine/threonine-protein kinase BUB1 beta*	520	119545	cytoplasm nucleus cytoskeleton	essential component of the mitotic checkpoint with kinase activity	237
**80.**	**P02686**	**MBP**	**Myelin basic protein** *	979	33117	peripheral membrane protein	formation and stabilization of myelin membrane	262
**81.**	**P60660**	**MYL6**	**Myosin light polypeptide 6**	446	16961	cytoskeleton	muscle protein	213
**82.**	**P35749**	**MYH11**	Myosin-11*	542	227339	Cytoskeleton and cytosol	muscle contraction	227
**83.**	**Q9UKX3**	**MYH13**	Myosin-13*	553	223605	cytoplasm	muscle contraction	693
**84.**	**Q8WXH0**	**SYNE2**	Nesprin-2*	526	796442	ubiquitous	involved in the maintenance of nuclear organization and structural integrity	191
**85.**	**Q8NF91**	**SYNE1**	Nesprin-1*	537 1011086		Nuclear cytoplasm cytoskeleton and membrane	involved in the maintenance of nuclear organization and structural integrity	222
**86.**	**Q9ULB1**	**NRXN1**	Neurexin-1*	561	161883	cell membrane	cell surface protein involved in cell-cell-interactions exocytosis of secretory granules and regulation of signal transmission	416
**87.**	**Q8NFP9**	**NBEA**	Neurobeachin*	578	327822	cytoplasm and peripheral membrane	protein localization anchoring/targeting kinase A to the membrane	359
**88.**	**Q6KC79**	**NIPBL**	**Nipped-B-like protein** *	809	316051	nucleus	involved in sister chromatid cohesion	236
**89.**	**P04198**	**MYCN**	N-myc proto-oncogene protein*	545	49561	nucleus	transcription factor proto-oncogene	146
**90.**	**P23497**	**SP100**	**Nuclear autoantigen Sp-100**	483	53768	nucleus and cytoplasm	transcription regulation and tumor suppressor	168
**91.**	**Q15788**	**NCOA1**	Nuclear receptor coactivator 1*	583	156757	nucleus	binds nuclear receptors and stimulates the transcriptional activities in a hormone-dependent fashion Involved in the coactivation of different nuclear receptors and mediated by STAT3 STAT5A STAT5B and STAT6 transcription factors	292
**92.**	**O00482**	**NR5A2**	Nuclear receptor subfamily 5 group A member 2*	808	61331	nucleus	transcription regulation	237
**93.**	**Q5VST9**	**OBSCN**	Obscurin*	569	868484	cytoplasm	involved in miofibrillogenesis	225
**94.**	**Q9C0B5**	**ZDHHC5**	**Palmithoyltransferase ZDHHC5**	917	77545	cell membrane	acyltrasferase	202
**95.**	**P54317**	**PNLIPRP2**	**Pancreatic lipase-related protein 2**	527	51947	secreted	lipid metabolism and degradation	173
**96.**	**Q8NG07**	**PNMA1**	**Paraneoplastic antigen Ma1**	478	39761	nucleus and cytoplasmic in tumor cells	paraneoplastic antigen	408
**97.**	**O15018**	**PDZD2**	PDZ domain-containing protein*	818	280092	nucleus cytoplasm and endoplasmic reticulum	cell adhesion	381
**98.**	**O95613**	**PCNT**	Pericentrin*	540	378037	cytoplasm	protein binding	447
**99.**	**Q5VV67**	**PPRC1**	Peroxisome proliferator-activated receptor gamma coactivator-related protein 1*	611	177544	nucleus	acts as a coactivator during transcriptional activation of nuclear genes related to mitochondrial biogenesis and cell growth	176
**100.**	**O00541**	**PES1**	**Pescadillo homolog 1**	693	68003	nucleus	ribosome biogenesis and rRNA processing	189
**101.**	**P15259**	**PGAM2**	**Phosphoglycerate mutase**	899	28766	nucleus cytosol	involved in glycolysis and gluconeogenesis	316
**102.**	**P16284**	**PECAM1**	**Platelet endothelial cell adesion molecular**	655	82536	cell membrane	protein binding	399
**103.**	**Q9HAU0**	**PLEKHA5**	Pleckstrin homology domain-containing family A member 5*	720	127464	cytoplasm	protein binding	294
**104.**	**Q15149**	**PLEC**	Plectin*	574	531791	cytoplasm	ankyrin binding and apotoptic process	258
**105.**	**Q9NS40**	**KCNH7**	Potassium voltage-gated channel subfamily H member 7*	757	135000	membrane	pore-forming (alpha) subunit of voltage-gated potassium channel	067
**106.**	**Q7L014**	**DDX46**	**Probable ATP-dependent RNA helicase DDX46**	933	117362	nucleus	nucleoside nucleotide and nucleic acid metabolism	178
**107.**	**Q7Z7M1**	**ADGRD2**	Probable G-protein coupled receptor 144*	833	104087	cell membrane	G-protein coupled receptor transducer	681
**108.**	**P35232**	**PHB**	**Prohibitin**	557	29804	Membrane and cytoplasm	DNA replication cell proliferation and differentiation proto- oncogene	347
**109.**	**P27918**	**CFP**	**properdin**	833	51276	Secreted	immunity and defense	497
**110.**	**Q13258**	**PTGDR**	Prostaglandin D2 receptor*	939	40271	cell membrane	receptor for prostaglandin D2	282
**111.**	**Q9P2B2**	**PTGFRN**	Prostaglandin F2 receptor negative regulator*	616	98556	endoplasmic reticulum membrane	protein binding	213
**112.**	**P14921**	**ETS1**	Protein C-ets-1*	503	50408	nucleus and cytoplasm	transcription factor	352
**113.**	**P80511**	**S100A12**	Protein S100-A12*	581	10575	Cytoplasm and cell membrane	signal transduction inflammatory processes and immune response	341
**114.**	**A3KN83**	**SBNO1**	Protein strawberry notch homolog 1*	796	154312	nucleus	regulation of transcription	412
**115.**	**Q13882**	**PTK6**	Protein-tyrosine kinase 6*	656	51834	cytoplasm and nucleus	involved in protein metabolism and modification implicated in the regulation of a variety of signaling pathways that control the differentiation and maintenance of normal epithelia as well as tumor growth	479
**116.**	**Q9Y315**	**DERA**	**Putative deoxyribose-phosphate aldolase** *	908	35231	cytoplasm	lyase	189
**117.**	**Q15311**	**RALBP1**	RalA-binding protein 1*	568	76063	membrane	signal transduction and ATP catabolic process	213
**118.**	**Q08999**	**RBL2**	Retinoblastoma-like protein 2*	727	128367	nucleus	transcription factor	338
**119.**	**Q7Z5J4**	**RAI1**	Retinoid-acid induced protein 1*	903	203352	cytoplasm and nucleus	transcriptional regulator	229
**120.**	**Q5T5U3**	**ARHGAP21**	**Rho GTPase-activating protein 21**	785	217331	peripheral membrane protein	GTPase-activating protein	248
**121.**	**Q9BST9**	**RTKN**	**Rhotekin**	718	62667	nucleoplasm	mediates Rho signaling to activate NF-kappa-B and increases resistance to apoptosis	276
**122.**	**Q14137**	**BOP1**	**Ribosome biogenesis protein BOP1**	580	83630	nucleus	ribosome biogenesis, rRNA processing	489
**123.**	**Q9H7B2**	**RPF2**	Ribosome production factor 2 homolog*	1000	35583	nucleus	poly(A) RNA binding	451
**124.**	**Q8WV20**	**RBMS1**	**RNA binding motif single stranded interacting protein 1**	891	44505	nucleus	nucleoside nucleotide and nucleic acid metabolism	607
**125.**	**P21817**	**RYR1**	Ryanodine receptor 1*	518	565176	sarcoplasmic reticulum membrane	calcium transport	484
**126.**	**O14641**	**DVL2**	Segment polarity protein dishevelled homolog DVL-2*	567	78948	cell membrane and cytoplasm	Wnt signaling pathway	379
**127.**	**Q99719**	**SEPT5**	**Septin-5**	621	42777	cytoplasm	GTO and protein binding	249
**128.**	**Q9UQ35**	**SRRM2**	Serine/arginine repetitive matrix protein 2*	1205	299615	nucleus	pre-mRNA processing and mRNA splicing	237
**129.**	**P15056**	**BRAF**	Serine/Threonine protein kinase B-raf*	729	84437	nucleus and cytoplasm	proto-oncogene	394
**130.**	**Q06190**	**PPP2R3A**	**Serine/threonine-protein phosphatase 2A regulatory subunit B’’ subunit alpha**	509	130278	Colocalized with protein phosphatase type 2A complex	calcium ion and protein binding and regulator of Wnt signaling pathway	290
**131.**	**P42345**	**MTOR**	Serine/threonine-protein kinase mTOR*	673	288892	ubiquitous	it is a central regulator of cellular metabolism growth and survival in response to hormones growth factors nutrients energy and stress signals	257
**132.**	**Q96Q15**	**SMG1**	Serine/threonine-protein kinase SMG1*	603	410501	nucleus and cytoplasm	kinase involved in mRNA surveillance and genotoxic stress response pathways	379
**133.**	**Q15464**	**SHB**	**SH2 domain-containig adapter protein B**	910	55042	cytoplasm	involved in angiogenesis and apoptosis	225
**134.**	**Q9H1V8**	**SLC6A17**	**Sodium-dependent neutral amino acid transporter SLC6A17**	568	81001	cytoplasmic vesicle multi-pass membrane protein	neurotransmitter transporter	201
**135.**	**Q96BI1**	**SLC22A18**	**Solute Carrier Family 22 member 18**	662	13354	cell membrane	zinc ion binding	236
**136.**	**O94956**	**SLCO2B1**	Solute carrier organic anion transporter family member 2B1*	870	76711	cell membrane	ion transport	171
**137.**	**P11277**	**SPTB**	Spectrin beta chain erythrocytic*	515	246468	cytoplasm	cell structure and motility	127
**138.**	**Q9BPZ7**	**MAPKAP1**	**Stress-activated map kinase interacting protein 1**	724	59123	cell membrane and nucleus	stress response and phosphatidic acid binding	463
**139.**	**Q15431**	**SYCP1**	Synaptonemal complex protein 1*	578	114192	Nucleus and chromosome	cell cycle and meiosis	342
**140.**	**Q9BQ70**	**TCF25**	Transcription factor 25*	595	76667	nucleus	transcriptional repressor	406
**141.**	**Q01664**	**TFAP4**	**Transcription factor AP-4**	563	38726	nucleus	transcription regulator	348
**142.**	**Q8NHW3**	**MAFA**	Transcription factor mammalian MafA*	749	36982	nucleus	transcriptional factor	941
**143.**	**Q8NEM7**	**SUPT20H**	**Transcription factor SPT20 homolog**	877	85789	nucleus	required for MAP kinase p38 (MAPK11 MAPK12 MAPK13 and/or MAPK14)	421
**144.**	**P29084**	**GTF2E2**	**Transcription initiation factor IIE subunit beta**	966	33044	nucleus	basal transcription factor	227
**145.**	**O75410**	**TACC1**	Transforming acidic coiled-coil-containing protein 1*	481	87794	cytoplasm and nucleus	cell cycle and division	555
**146.**	**Q01995**	**TAGLN**	Transgelin*	887	22611	cytoplasm	muscle protein	099
**147.**	**Q9UJA5**	**TRTM6**	**tRNA (adenine(58)-N(1))-methyltransferase non-catalytic subunit TRM6**	718	55799	nucleus	tRNA processing	205
**148.**	**Q9NYL9**	**TMOD3**	**Tropomodulin-3**	508	39595	cytoplasm	blocks the elongation and de-polymerization of the actin filaments	122
**149.**	**P06753**	**TPM3**	**Tropomyosin alpha-3-chain**	468	32950	cytoplasm and cytoskeleton	muscle protein	294
**150.**	**P07951**	**TPM2**	**Tropomyosin beta chain**	466	32851	cytoplasm and cytoskeleton	muscle protein	067
**151.**	**P49815**	**TSC2**	Tuberin*	698	200608	cytoplasm	tumor suppressor and intracellular protein traffic	401
**152.**	**P07437**	**TUBB**	Tubulin beta chain*	478	49671	cytoplasm and cytoskeleton	protein binding and structural constituent of cytoskeleton	435
**153.**	**P78324**	**SIRPA**	Tyrosine-protein phosphatase non-receptor type substrate 1*	651	54967	membrane	involved in intracellular signaling during synaptogenesis and in synaptic function	276
**154.**	**Q9NPG3**	**UBN1**	Ubinuclein-1*	937	121520	nucleus, cell junction	novel regulator of senescence	262
**155.**	**Q14139**	**UBE4A**	Ubiquitin conjugation factor E4 A*	511	123522	cytoplasm	protein metabolism and modification	261
**156.**	**Q9Y6A4**	**CFAP20**	Cilia- and flagella-associated protein 20*	978	22774	nucleus	transcription factor	093
**157.**	**Q15849**	**SLC14A2**	Urea transporter 2*	651	101209	cell membrane	transport protein	127
**158.**	**Q8N6Y0**	**USHBP1**	**Usher syndrome type-1C protein-binding protein 1**	558	76068	cytoplasm nucleus plasma membrane	signal transduction	169
**159.**	**P62955**	**CACNG7**	**Voltage-dependent calcium channel gamma 7 subunit**	665	31003	membrane	calcium transport	346
**160.**	**P21281**	**ATP6V1B2**	**V-type proton ATPase subunit B brain isoform**	557	56501	peripheral membrane protein	cation transport	148
**161.**	**Q9UJW8**	**ZNF180**	Zinc finger protein 180 (HHZ168)*	804	79111	nucleus	involved in transcriptional regulation	193
**162.**	**Q7Z3V5**	**ZNF571**	Zinc finger protein 571*	871	70792	nucleus	involved in transcriptional regulation	354
**163.**	**Q9H582**	**ZNF644**	**Zinc finger protein 644**	843	149565	nucleus	involved in transcriptional regulation	369
**164.**	**Q15776**	**ZKSCANS**	**Zinc finger protein with KRAB and SCAN domains 8**	704	65816	nucleus	transcription factor	312

a The identification and quantitation of proteins were performed using the Protein Pilot Paragon Method The MS/MS data were processed using a mass tolerance of 10 ppm and 02 Da for the precursor and fragment ions respectively

b According to “UniProtKB” (http://wwwuniprotorg/)

c According to “Compute pI/MW” (http://webexpasyorg/compute_pi/)

* Proteins involved in epithelial neoplasia (p-value=362E-05).

The input data set containing all identified proteins from the iTRAQ LC−MS/MS analysis was uploaded into IPA [19]. The founded top five significant Molecular and Cellular Function associations with proteins are involved in *Cellular Movement*, *Cellular Assembly* - *Organization*, *Cellular Development*, *Cellular Growth - Proliferation*, and *Gene Expression*. Otherwise the top five obtained networks are all related to cellular proliferation, cellular death/survival and cancer (Supporting Information, Table SI3 A-F). IPA analysis evidenced that among all dataset, 105 molecules are involved in epithelial neoplasia with a p-value of 3.62E-05 (Table 1).

## DISCUSSION

A crucial step in cancer control and prevention is the detection of disease as early as possible in order to allow effective interventions and therapies. Biomarkers are important as molecular signposts of the physiological state in specific cell at a definite time. In an effort to develop a comprehensive approach for biomarker-based prevention research it became primordial to draft a modern proteomic platform technology for biomarkers discovery and validation. Several studies have been focused on prostate cancer research through MS-based proteomic approaches [8] but biomarkers discovery remains a difficult task related to the complexity of the samples and the dynamic concentration of proteins. The mass spectrometry based proteomic approach described in this work is focused on the extraction, identification and quantitation of a base-soluble proteins subset from prostate tissue useful for diagnosis of human PCa. The choice for the analysis of stage-specific tumours (T) and healthy tissues adjacent to the tumour (NT) area could help in the elucidation of the molecular networks and mechanisms involved in pathogenesis. T and NT prostate tissue from the same individual were analysed since tissue samples show a wide biological variability particularly when they derive from different patients. The identification of basesoluble proteins could have the main advantage to be directly correlated to body fluids such as urine, which is enriched with proteins from PCa cells, hence giving the option to develop an alternative non-invasive biomarkers discovering method. The experimental design was planned to generate a consistent data set and to reduce the number of analytes handling, minimizing the result variability. The introduction of a pre-fractionation step prior to proteomic analysis reduce the sample complexity and improve the detection sensitivity of low-abundant proteins [20]. The buffers supplied by manufacture contain surfactants and salts that interfere with MALDI-TOF MS analysis, therefore we have developed a novel depletion protocol adopting saline solutions MS-compatible.

### Differentially expressed proteins

**Figure 2:**
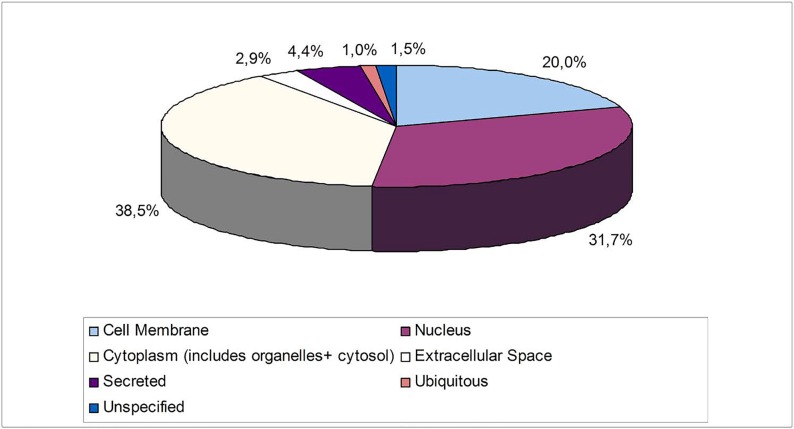
Functional distribution of the identified proteins in accordance to their cellular location

Table 1 lists 164 proteins that were identified and quantified by Protein Pilot Paragon methods. The identified proteins were grouped in different classes which were based on their cellular location (Figure 2). The major parts of the proteins originated from the cytoplasm (38,5%) and nucleus (31,7%). The presence of membrane related proteins (20,0%) confirms the high-throughput performance of the extraction step. The origins of the remaining proteins were as follows: secreted (4,4%), ubiquitous (1%) and -from extracellular space (2,9%), while only a small part (1,5%) was unspecified.

Table 1 list several proteins involved in transcriptional regulation. The transcription factors participate in the gene expression at the ends of all 19 of the know signal transduction and stress pathways. [21] An increase in the activity of the transcription factors is correlated with the various changes in the protein expression, protein stability, protein-protein interaction and post-translation modification [21]. The increase of many of these activities can affect the cancerous transformation by modifying the typical function of transcriptional co-activator or co-repressors. Among the family of the transcription receptor factor, the nuclear receptor coactivator 1 protein (NCOA1, Table 1 - row 91), also called SRC-1, identified as up-regulated,. SRC-1 is a co-activator of the androgen receptor (AR) mediated signalling pathway. The involvement of the NCOA1 in prostate cancer progression was supported by the recent study of Agoulnik et al. [22]. NCOA1 over expression in the metastatic prostate cancer occurs in primary tumors rather than the normal prostate. Agoulnik et al demonstrated that the ablation of NCOA1 in the androgendependent LNCaP prostate cancer cells, represses the activation of the AR target genes and it reduces the ARdependent cellular proliferation. Prohibitin (PHB, Table 1 - row 108) is an evolutionary conserved multifunctional protein that is upregulated in PCa samples and is also implicated in many cellular process [23, 24, 25]. Several studies have shown that the essential function of PHB is for cell proliferation and it as a crucial protein used for cancer cell growth and survival [26]. In accordance with our result, Umanni et al. [27]. examined biopsy

samples from benign prostate hyperplasia (BPH) and PCa patients proving a significant up-regulation of prohibitin in tumoral samples. A significant alteration change was observed in the expression of Actin and microtubule Cytoskeleton proteins (Table 1 - rows 3, 37, 38, 39, 63, 81, 82, 83, 137, 146,149, 150). These proteins are able to organize the cytoplasmic organelles and the intracellular compartments in order to drive the chromosomal separation and the cell division during morphogenesis, cell cycle, and to generate forces during cell migration [28, 29]. Myosin filaments (Table 1, rows 81, 82, 83, 149, 150) determine cell surface contractions and muscle cell contraction in accordance with actin. The kinesin (Table 1, rows 64, 65) and dynein (Table 1, rows 37, 38) proteins carry numerous cellular function including the transport of vesicles and organelles within cells, the beating of flagella and cilia and within the mitotic and meiotic spindles to segregate replicated chromosomes. Within this protein family, kinesin ensures a crucial role in the occurrence and development of human cancer. A great number of proteins from the kinesin super-family show abnormal over-expression in various cancer cells and this expression level indicates as prognostic marker for breast and lung cancer [30, 31]. A change of expression of the members of the G protein coupled receptor proteins is evident (GPRs, Table 1 rows 42, 107, 110). The GPRs belong to a family of cell-surface molecules implicated in signal transmission. GPRs proteins are implicated in many biological process as cell proliferation, motility, angiogenesis and metastasis and it has been recently highlighted the they are over expressed in various cancer type and have an incisive role to tumor cell growth [32]. The upregulated activity of GPRs might contribute to transition from hormone dependent to hormone independent tumor for prostate and breast cancer. Marinissen et al., [33] suggested that in PCa cell, GPRs can stimulate ERK phosphorylation and increase the transcription of ARs. The observed over regulation of kinases (Table 1, rows 26, 56, 59, 78, 79, 115, 129, 131, 132, 138) is fully in accordance with the data reported [34, 35]. In particular an oncogenic role was indicated for the non-receptor type tyrosine kinase, Protein Tyrosine Kinase 6 (PTK6, Table 1 row 115) [36]. PTK6 promotes cancer cell proliferation, migration and survival through activating oncogenic signalling pathways. Moreover it is involved in the activation of signal transducers and activators of transcription (STATs) that control tumorigenesis [37] and promotes AKT activation and phosphorylation [38]. Zheng et al. have described the increased levels of PTK6 mRNA in prostate cancer with respect to healthy normal prostate tissue and normal tissue adjacent to the tumor [39]. The same authors evidenced an higher expression of PTK6 in metastatic human prostate cancer samples, suggesting an oncogenic role for PTK6 in prostate tumor development and metastasis [40].

### Pathway and network analyses

**Figure 3:**
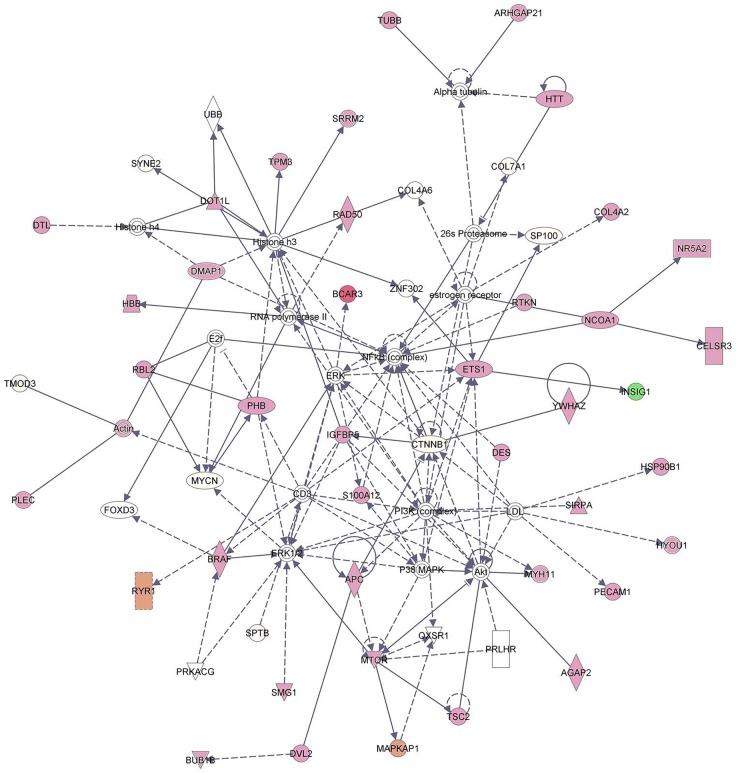
“Cell Death and Survival, Cancer, Gastrointestinal disease” network of 70 proteins observed de-regulated in tumoral prostate tissue by the iterative Ingenuity Pathway Analysis software program The node and edge represent the proteins and their interactions, respectively, while the intensity of the node color indicates degree of up- (red) or down- (green) regulation.

Proteomic data were analyzed using IPA software to select protein involved in cancer development, occurrence or progression and to evidence the biological processes in which these proteins are involved. IPA analysis suggests five Top Networks (Supporting Information, Table S3), the first one related to “Cell Death and Survival, Cancer” comprises 70 focus molecules and evidences as the majority of identified protein are directly and not mainly involved in three signalling pathways that play a crucial role in cancerogenesis: (i) the extracellular signal-regulated kinase (ERK) signaling pathway, (ii) the Nuclear factor kappa B (NF-ĸB) pathway and (iii) phosphatidylinositol 3-kinase/protein kinase-B/mammalian target of rapamycin (PI3K/AKT/mTOR) signalling cascade (Figure 3).

The extracellular signal-regulated kinase (ERK) signalling pathway controls a broad range of cellular activities such as proliferation, survival, differentiation and motility. ERK regulates chromatin remodelling through the phosphorylation of cytoplasmic and nuclear targets as transcriptional factors and Cytoskeleton proteins [41]. In addition, activation of ERK 1/2 due to radiation, osmotic stress or tumor necrosis factor (TNF) inhibits apoptosis, while inhibition of the same pathway supports apoptosis. It has been shown that the increased activity of extracellular signal-regulated kinase is implicated in the development and prognosis of PCa [42]. Nuclear factor kappa B (NF-κB) transcription factors regulate several important physiological processes, including inflammation and immune responses, cell growth, apoptosis, and the expression of certain viral genes. The NF-κB pathway is often active and plays a key role in the disease since it involves a sequence of transcription factors that stimulate promotion and progression of tumors as well as chemotherapy and radiotherapy resistance [43] and it is clear that modulators of this pathway can act at several levels [44]. The phosphatidylinositol 3-kinase/protein kinase-B/mammalian target of rapamycin (PI3K/AKT/mTOR) signalling cascade is a key oncogenic signalling pathway, which has a central role in several cellular processes significant for cancer progression [45]. The PI3K–AKT pathway is inappropriately activated in many cancers by receptor tyrosine kinases. PI3K/AKT/mTOR pathway prevents apoptosis, induce cancer cell growth and promotes resistance to anticancer therapies acting on cellular differentiation and metabolism [46, 47]. Recently, several researches have demonstrated that the activation of the PI3K/AKT/mTOR pathway was strongly implicated in the prostate cancer progression [48]. Moreover, Gao et al. suggested that this signalling pathway could serve as a novel target for therapeutic intervention in prostate cancer [49].

### PCa differentially expressed proteins vs bodily fluids

Proteomic data were further elaborated by IPA in order to maximize the impact of the information, to get a more comprehensive understanding about the obtained results and suggest the proposal of biomarkers to screening populations at risk for cancer. The device “Biomarker Filter” measures whether a particular protein is detectable in tissue or bodily fluids in an effort to identify a cohort of possible proteins associated with a specific disease. The proteomic data are evaluated by three restriction levels: (i) Urine, (ii) Urine and Prostate Gland, (iii) Urine, Prostate Gland and Plasma/Serum. Eleven up- and downregulated proteins are selected and reported in Table 2. These 11 proteins are eligible cancer biomarkers and are also present in a set of bodily fluids. In PCa Catenin Beta 1 (CTNNB1, Table 2) contributes to cadherin-mediated adhesion and acts as coactivator binding androgen receptor suggesting that it has a role in castration-resistant disease [50]. An abnormal activation of WNT/β-catenin signalling has been reported in colon cancer [51], and a typical upregulation of cytoplasmic β-catenin was detected in thyroid carcinogenesis [52]. The observed down-regulation of Tropomyosin 2 (TPM2, Table 2) is in agreement with several studies that proved the association of its altered expression with carcinogenesis [53]. The expression change of TPM isoforms can be induced by variety of carcinogens including chemical carcinogens, UV radiation, DNA and RNA tumor viruses during cancer cell transformation. Varisli showed that the expression of TPM2 may decrease with growing score of cancer and suggested the level of this protein are useful as a prognostic biomarker tool for prostate cancer [54]. The up regulation of tropomyosin alpha-3-chain (TPM3, Table 2) is supported by the results of Franzen et al. in which they have found higher level of TPM isoform in the primary breast cancer that had metastasised, rather than in the axillary lymph nodes [55].

**Table 2:** Proteins from prostatic gland that are also present in bodily fluids^a^

Gene Name^(a)^	Accession N^(b)^	Entrez Gene Name	Location	Family	Fold Change	Blood	Plasma/Serum	Urine	Prostate Gland
**BRAF**	**P15056**	v-raf murine sarcoma viral oncogene homolog B ^(e)^	Cytoplasm	kinase	394	x	x		x
**DPYS**	**Q14117**	Dihydropyrimidinase ^(c)^	Cytoplasm	enzyme	6304			x	
**CTNNB1**	**P35222**	catenin (cadherin-associated protein) beta 1 88kDa ^(e)^	Nucleus	transcription regulator	2112	x			x
**IGFBP5**	**P24593**	insulin-like growth factor binding protein 5 ^(d)^	Extracellular Space	other	3065			x	x
**MTOR**	**P42345**	mechanistic target of rapamycin (serine/threonine kinase) ^(e)^	Nucleus	kinase	257	x	x		x
**PGAM2**	**P15259**	phosphoglycerate mutase 2 ^(d)^	Cytoplasm	phosphatase	316			x	x
**PECAM1**	**P16284**	platelet/endothelial cell adhesion molecule 1 ^(cde)^	Plasma Membrane	other	399	x	x	x	x
**TAGLN**	**Q01995**	Transgelin ^(cde)^	Cytoplasm	other	−1002			x	x
**TPM3**	**P06753**	tropomyosin alpha-3-chain ^(cd)^	Cytoplasm	other	2940			x	x
**TPM2**	**P07951**	tropomyosin 2 (beta) ^(e)^	Cytoplasm	other	−1484	x	x		x
**YWHAZ**	**P63104**	tyrosine 3-monooxygenase/tryptophan 5-monooxygenase activation protein zeta ^(cd)^	Cytoplasm	enzyme	3806	x		x	x

a According to QUIAGEN ‘s Ingenuity^®^ Pathway Analysis - Biomarker Filter

b According to “UniProtKB” (http://wwwuniprotorg/) In the table are listed proteins markers suggested by IPA when Biomarker Filter is restricted to

c Urine

d Urine and Prostate Gland

e Urine Prostate Gland Blood and Plasma/Serum.

Up-regulation of the tyrosine 3-monooxygenase/tryptophan 5 monooxygenase activation protein zeta (YWHAZ, Table 2), a 14-3-3 zeta isoform., belonging to the 14-3-3 protein family, was observed. In humans, 7 different 14- 3-3 isoforms have been identified ubiquitously expressed and highly conserved in all eukaryotic organisms [56]. This protein family interact with hundreds of binding partners and is involved in the regulation of vital cellular processes [57]. 14-3-3 protein family was associated with proto-oncogene and oncogene products suggesting a direct contribute to cancer development [58]. Murata et al. [59] analyzed the immunoreactivity of YWHAZ in formalin fixed paraffin embedded sections of benign and tumoral prostate tissue evidencing the protein overexpression in PCa tissue. Platelet endothelial cell adhesion molecule-1 (PECAM-1, Table 2) is a 130kDa membrane glycoprotein belonging to the immunoglobulin superfamily that is able to mediate both homophilic and heterophilic adhesions. PECAM-1 appears to be involved in a variety of biological functions. [60] Karagianis et al. found the up-regulation of PECAM-1 of the proteome of endothelial cells, in which PECAM was differentially regulated by an androgenindependent angiogenic response [61]. The down regulation of Transgelin (TAGLN, Table 2), is consistent with several studies which reported significantly lower levels of TAGLN expression in the immortalised human prostate epithelial cell line RWPE-1, in the metastatic LNCaP cells and in the metastatic PC3 [62]. The down regulation of transgelin can be correlated to the prostate cancer progression, it may be used as a marker for cancer in addition to provide a target for novel cancer therapies. Perturbation of PTK signalling by mutations and other genetic alterations results in deregulated kinase activity and malignant transformation. It well know the switch role of the mammalian target of rapamycin, mTOR (Table 2), in regulating life or death signals, between “cell growth - cell cycle” and “damaged microtubules”. mTOR is emerged as a critical effector in cell-signaling pathways commonly deregulated in human cancers suggesting that mTOR inhibitors may be useful in oncology [63]. BRAF is a serine/threonine kinase (Table 2) that is commonly activated by somatic point mutation in human cancer and his activity is also regulated by phosphorylation of residues in the activation segment. Moreover the high frequency of mutations in melanoma and the relative lack of effective therapies suggested that inhibition of BRAF activity may be an important new strategy in the treatment of some cancer types [64]. The upregulation of Dihydropyrimidinase enzyme (DPYS, Table 2) is another important data. DPYS deficiency induces haematological or gastrointestinal toxicity during treatment with 5-fluorouracil for common neoplasms [65]. Pyrimidine pathways are fundamental in human physiology and several studies report their upregulation in malignancy [66] making them ideal targets for pharmacological intervention. Finally, the identification of upregulated insulin-like growth factor binding protein 5 (IGFBP5, Table 2) is in agreement with its role in the IGF system, where is involved in normal growth and development. In particular increased expression of IGFBP5 has been reported in tumors of the gastrointestinal tract [67, 68]. IGFBP5 appears to exert a specific inhibitory effect on melanoma growth and metastasis through inhibition of the ERK1/2 and P38-MAPK pathways, therefore it may qualify as a useful therapeutic target against melanoma and other cancers [67].

The proposed proteomic approach, focused on base-soluble proteins from tissue and present in biological fluids, constitutes a study leading to a deeper understanding of the PCa from a molecular perspective. The selective proteome extraction allows a direct correlation and identification of deregulated pathways providing a panel of candidate diagnostic biomarkers. A limitation of the study might be the relatively small sample number, but the opportunity to transfer this results on other biological matrices, more easily available (as body fluids), opens new chances. The identification of eleven deregulated proteins from prostatic gland, present in body fluids, and some specific for urine, could be an important start point to select new cancer biomarkers. Further studies are needed to confirm the proposed biomarkers and to evaluate the diagnostic potential of the other differentially expressed proteins which might further improve the diagnostics accuracy of the proposed set.

## MATERIALS AND METHODS

### Reagents and chemicals

Ammonium Bicarbonate (NH_4_HCO_3_, 99.5%), trypsin (proteomics grade), α-cyano-4-hydroxy-transcynnamic acid (α-CHCA, 99,0%), water (HPLC grade), trifluoracetic acid (TFA, 99,0%), methanol (HPLC grade), acetone, protease inhibitor cocktail and protein standards for protein molecular weight marker were purchased from Fluka-Sigma Aldrich S.r.l. (Milan, Italy). Protein standards and reagent for protein quantification were acquired by Bio-Rad's Laboratories, Inc. (Monza, Italy). iTRAQ reagents and buffers were obtained from Applied Biosystems (Foster City, CA). Peptide and protein standards, for mass spectrometer external calibration, were prepared from the Sequazime peptide mass standard kit (Applied Biosystems, Framingham, MA, USA).

### Protein extraction

The experimental procedure was developed on porcine prostate tissue. The prostate tissue was given by official slaughterhouse after veterinary inspection and transferred in ice in laboratory. Tissues were washed three times in ice-cold phosphate buffered saline, cut in small pieces, weighed and freezed at −80°C until the protein extraction. The tissues obtained from a total of ten patients (A-L) affected by prostate cancer (Gleason score 6, 3+3) with elevated PSA level (between 10 to 19 ng/ml), classified by Tumour Node Metastasis (TNM) as T1c, N0, M0, were selected for the study after informed consent. This study was approved by the ethics committee of Magna Graecia University, patients had signed a written consent to prostate biopsies and clinical data access for research purpose. After radical prostatectomy “Non Tumoral” (NT) and “Tumoral” (T) fragments prostate tissue from the same individual were cut in two sections. One section was formalin fixed paraffin embedded and stained with hematoxylin-eosin for histological evaluation while the second one was immediately frozen at −80°C prior to proteins extraction. The frozen prostate tissue were powdered in liquid nitrogen. The powdered tissues were further homogenized in 1 mL of a cold solution containing 50mM NH_4_HCO_3_ (pH 8), 0,05% SDS (v/v) and protease inhibitor cocktail (1:100, v/v), then submitted to sonication conditions 3 times for 10s/time [17, 18]. Each operation was performed on ice. The resulting homogenates were centrifuged at 50,000 × g for 1h at 4°C. Concentration of protein extracted was determined by Bradford's assay [69].

### Immunodepletion of high-abundant proteins

The porcine proteins extracted were depleted of high abundant proteins using two commercially cartridge: “Multiple affinity removal spin cartridge” (Agilent Technologies, Milan, Italy, 5188-5230) and “ProteoPrep Blu Albumin and IgG depletion Medium” (Sigma Aldrich, PROT-BA). The cartridge were treated three times with 200 μl of 50mM NH_4_HCO_3_, (pH 8), before loading the sample. A volume of 200 μl, containing 500 μg of extracted proteins, were applied on column and incubated for 10 min at room temperature. After centrifugation at 3000 rpm for 1 min, the flow-through fraction (depleted of albumin, IgG, IgA, transferrin, haptoglobin and α1-antitrypsin for Agilent column and of albumin and IgG for Sigma column) were loaded again on column, centrifuged and collected. The cartridges were washed two times with 200 μl of 50mM NH_4_HCO_3_ and the relative flow-through were collected and combined with the previous depleted fractions. To elute the membrane-bound high abundant proteins, two washing with (NH_4_)_2_CO_3_ (pH 10), were performed. After 10 min of incubation and a subsequent centrifugation at 3000 rpm for 2 min, the eluted fractions were collected. An aliquot of low abundant proteins fraction and of high abundant eluted proteins were analyzed directly by linear MALDI mass spectrometry and the relative protein amount was quantified by Bradford's assay. Moreover, each fraction eluted was visualized on SDS-PAGE. Depletion of high abundant proteins for human prostate was performed only with Multiple affinity removal spin cartridge.

### SDS-page

Depleted flow-through, eluted fraction containing high abundant proteins and an aliquot of whole extracted proteins were analyzed by SDS-PAGE. All fractions were mixed with 5x gel loading buffer, containing 2-mercaptoethanol and bromophenol blue, denaturated at 95°C for 10 min before electrophoresis analysis in 12.5% sodium dodecyl sulphate-polyacrylamide gel electrophoresis (SDS-PAGE). Precision Plus Protein kaleidoscope standard (Bio-Rad's Laboratories, Milan, Italy) was loaded in the molecular weight marker lane for porcine samples, while an homemade protein molecular weight marker (Lactoferrin 87 kDa, L9507; Bovine Serum Albumin 66 kDa, A2153; Albumin from chicken 44 kDa, A5503; Mioglobin from equine skeletal muscle 17 kDa, M0630; Cytocrome C 12 kDa, C2506) was adopted for human proteins. Proteins were stained with Comassie Brillant Blu R-250 for 4 hours and destained overnight with a solution containing 40% MeOH, 10% CH_3_COOH and 50% H_2_O.

### Porcine protein digestion

Fifty micrograms of pig prostatic proteins from the depleted fraction proteins were digested overnight with trypsin, protein to enzyme ratio of 20:1, at 37°C in NH_4_HCO_3_, 50mM (pH 8.0) and dried by Concentrator Plus system (Eppendorf, Hamburg, Germany).

### Human proteins digestion and iTRAQ sample Labelling

The experimental conditions for i-TRAQ quantitative analysis were modified as follows. The six standard proteins mixture was digested with trypsin (ratio enzyme: substrate, 1:20) in a solution of Tetraethylammonium bromide (TEAB, 0.5M) and labelled without alkylation and reduction steps. The resulting peptides mixture was separated by off line RP-HPLC and analysed by MALDI-TOF MS. Approximately 40-60% of Six-protein Mix peptides were identified and quantified. 20 peptides of Bovine Serum Albumin (P02769), 23 peptides of β-Galactosidase (P00722), 2 peptides of α-Lactalbumin (P00711), 4 peptides of β-Lactoglobulin (P02754), 4 peptides of Lysozyme (P00698) and 18 peptides of Apotransferrin (P02787) were identified by MS/MS analysis (Table S1, Supporting Information). The number of identified peptides was satisfactory for the unique protein identification with suitable sequence coverage.

Two hundred micrograms of proteins from immunodepleted fractions were precipitated overnight at −20°C in six volume of cold acetone. The pellet was re-suspended in 30 μl of 500mM triethyl ammonium bicarbonate buffer (TEAB, supplied by Applied Biosystem and named as “Dissolution Buffer”) and the proteins were quantified by Bradford's Protein Assay. Ten micrograms of each NT fraction from patients A-L were pooled together and digested with trypsin, protein to enzyme ratio of 20:1, at 37°C overnight. The same procedure was performed for T fractions from patients A-L. Tryptic peptides were labelled with the iTRAQ reagents (m/z 115.1 and 117.1) following the manifacturer's protocol (Applied Biosystem). Briefly, the iTRAQ reagents were thawed at room temperature and spun to collect the reagent at the bottom of the tube and dissolved in 70μL of ethanol. The iTRAQ labels were added to the digested samples, in particular m/z 115.1 reporter ions was added to NT sample, while m/z 117.1 to T samples. The mixture was vortexed, centrifuged and incubated for 90 min on a rocker at 5rpm (Digital Rocker RK-1D, Witeg, Germany). The labelled samples were combined and dried in Concentrator Plus system prior to reverse phase chromatography [70–72] (RP-HPLC) fractionation as reported.

### MALDI-TOF MS and MS/MS analysis

Linear MALDI-TOF spectra were acquired with a 4700 Proteomics Analyzer mass spectrometer from Applied Biosystems (Foster City, CA) equipped with a 200-Hz Nd:YAG laser at 355-nm wavelength. A 1-μL portion of a premixed solution of whole or depleted samples and α-CHCA (0.3% in TFA) was spotted on the matrix target, dried at room temperature, and analyzed in the mass spectrometer. Spectra were acquired averaging 2500 laser shots with a mass accuracy of 500 ppm in default calibration mode that was performed using the following set of standards: insulin (bovine, [M + H]+ average m/z 5734.59), apomyoglobin (horse, [M +H]2+ average m/z 8476.78, [M + H]+ average m/z 16 952.56), and thioredoxin (Escherichia coli, [M + H]+ average m/z 11 674.48). MS and MS/MS analysis of offline spotted peptide samples were performed using the 5800 MALDITOF/TOF analyzer (AB SCIEX, Darmstadt, Germany) equipped with a neodymium: yttrium-aluminiumgarnet laser (laser wavelength: 349 nm), in reflectron positive-ion mode. All chromatographic fractions were re-suspended in 10 μl of α-CHCA matrix (10 mg/mL, CH_3_CN/0,3% TFA in water, 50:50, v:v), 1 μl of peptides matrix mixed solution was spotted on a MALDI plate and dried at room temperature. At least 4,000 laser shots were typically accumulated with a laser pulse rate of 400 Hz in the MS mode, whereas in the MS/MS mode spectra up to 5,000 laser shots were acquired and averaged with a pulse rate of 1,000 Hz. MS/MS experiments were performed at a collision energy of 1kV and ambient air was used as the collision gas with a medium pressure of 10^−6^ Torr. Protein identification was performed with the Protein Pilot 4.0 software program (AB Sciex) using the Paragon protein database search algorithm (AB Sciex).^20^ The data analysis parameters for porcine samples were: Sample Type: Identification; Cys Alkylation: None; digestion: Trypsin; Instrument: 5800 AB Sciex; Species: Suis Scrofa; Database: SwissProt; Search Effort: Thorought ID; Detected Protein Threshold [unused Protscore (Conf)]:1.5 (95,0%). For human labelled proteins, the data analysis parameters were as follows: Sample type: iTRAQ 4plex (Peptide Labelled); Cys Alkylation: None; Digestion: Trypsin; Instrument: 5800; Special Factors: Phosphorylation emphasis, Species: Homo Sapiens; Quantitated tab: checked; ID Focus: Biological modification and Amino acid substitutions; Database: SwissProt_UniProt; Search Effort: Thorough ID; Minimum Detected Protein Threshold [Unused ProtScore (Conf)]: 1.3 (95.0%); Run False Discovery Rate Analysis Tab: checked. The relative quantification was based on the ratio of the reporter ions corresponding to the T tryptic peptides (117.1 Da) over the ratio of the NT (115.1 Da) reporter ions. Proteins giving tryptic peptides with an average reporter ion ratio ≥2 were classified as up-regulated, otherwise those with an average reporter ion ratio ≤0.5 were classified as downregulated [8]. All identified proteins were analyzed through the use of QUIAGEN ‘s Ingenuity^®^ Pathway Analysis (IPA^®^, QUIAGEN Redwood City, www.quiagen.com/ingenuity).

## SUPPLEMENTARY FIGURES AND TABLES




