# Supplementary material for: Targeted proteomic approach in prostatic tissue: a panel of potential biomarkers for cancer detection
Source: Oncoscience. 2016 Jul 8;3(7-8):220–41. doi: 10.18632/oncoscience.313 (PMC5043072; doi:10.18632/oncoscience.313)
Supplement: Supplementary file 1 [file oncoscience-03-220-s001.pdf]

Targeted proteomic approach in prostatic tissue: a panel of potential biomarkers for cancer detection

SUPPLEMENTARY FIGURES AND TABLES

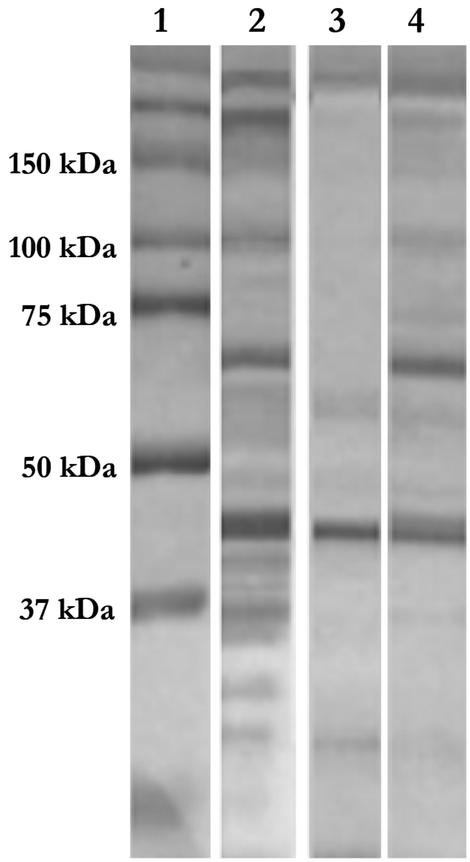

Supplementary Figure S1: Electrophoresis profile of prostate porcine tissue. Lanes: 1. Marker. 2: Whole tissue proteins extracted. 3: Depleted fraction from Multiple affinity removal spin cartridge (Agilent Technologies). 4: Depleted fraction ProteoPrep Blu Albumin and IgG depletion Medium (Sigma Aldrich).

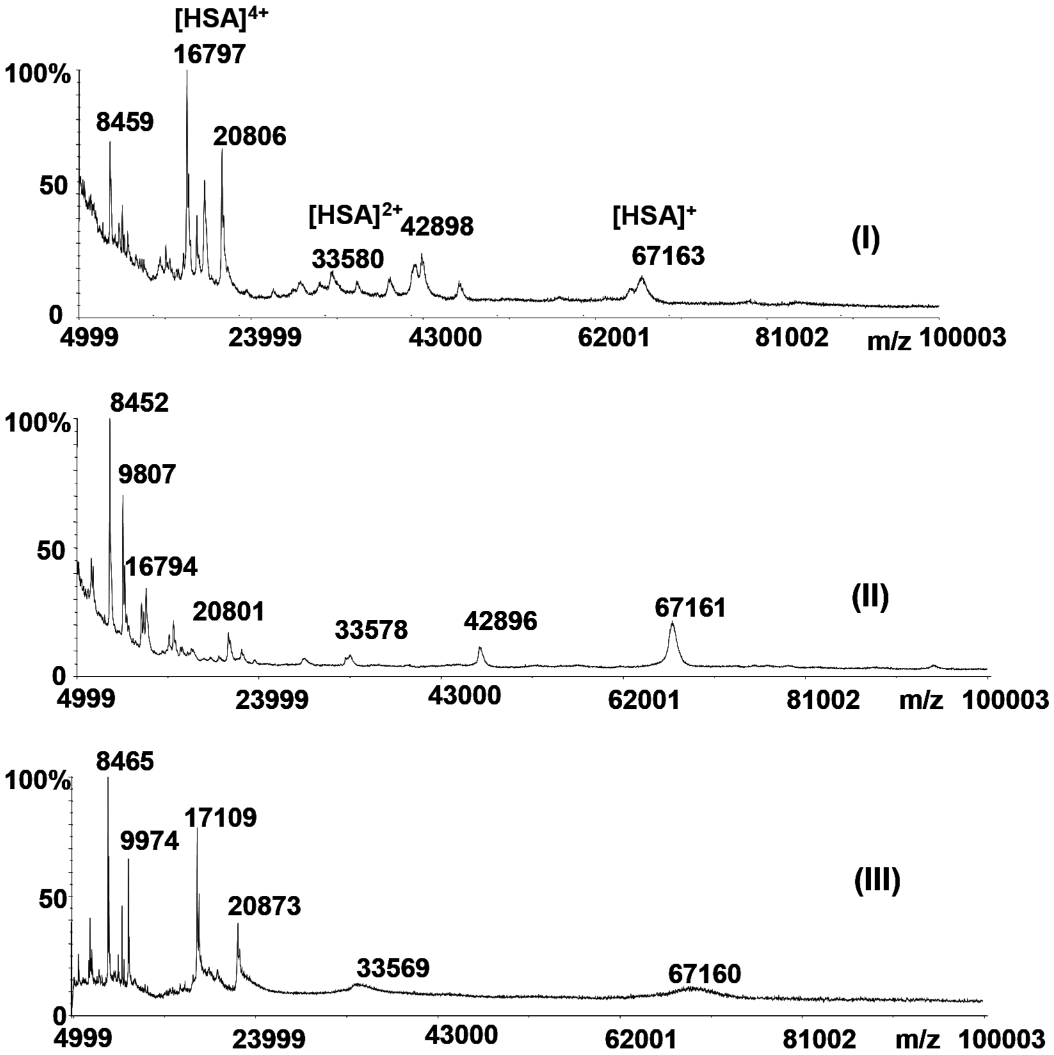

Supplementary Figure S2: Linear MALDI spectra of prostate human tissue from patient B. MALDI MS spectra of (I) whole saline protein extracted from NT tissue, (II) whole saline protein extracted from T tissue, (III) hLA after "Multiple affinity removal spin cartridge" - Agilent Technologies from T tissue.

Supplementary Table S1: Identified six protein-mix peptides

See Supplementary File

Supplementary Table S2: Identified proteins and peptide sequences from tumoral and non tumoral prostate tissue by MS/MS data processing

See Supplementary File

Supplementary Table S3: Ipa data set

See Supplementary File
