# Supplementary material for: Targeted proteomic approach in prostatic tissue: a panel of potential biomarkers for cancer detection
Source: Oncoscience. 2016 Jul 8;3(7-8):220–41. doi: 10.18632/oncoscience.313 (PMC5043072; doi:10.18632/oncoscience.313)
Supplement: Supplementary file 2 [file oncoscience-03-220-s002.pdf]

Table 1S: Identified Six Protein-Mix peptides

	Sequence [a]	*Num. of Labels*	*Calculated [MH]⁺ labeled* [c]	*Found [MH]⁺ labeled* [c]
	**Bovine Serum Albumin (P02769)** [b]			
**1.**	YLYEIAR	1	1071.60	1071.61
**2.**	ATEEQLK	2	1106.64	1106.65
**3.**	LVVSTQTALA	1	1146.69	1146.70
**4.**	AEFVEVTK	2	1210.70	1210.71
**5.**	CCTESLVNR	1	1260.54	1260.54
**6.**	NECFLSHK	2	1311.65	1311.66
**7.**	EACFAVEGPK	2	1384.69	1384.70
**8.**	HPEYAVSVLLR	1	1427.84	1427.83
**9.**	LVNELTEFAK	2	1451.84	1451.85
**10.**	LGEYGFQNALIVR	1	1623.90	1623.91
**11.**	VPQVSTPTLVEVSR	1	1655.95	1655.96
**12.**	SLHTLFGDELCK	2	1696.87	1696.89
**13.**	DAFLGSFLYEYSR	1	1711.85	1711.86
**14.**	ETYGDMADCCEK	2	1744.67	1744.68
**15.**	EYATLEECCAK	2	1768.76	1768.77
**16.**	DDPHACYSTVFDK	2	1831.83	1831.84
**17.**	ECCHGDLLECADDR	1	1860.67	1860.68
**18.**	YNGVFQECCQAEDK	2	2013.85	2013.86
**19.**	LFTFHADICTLPDTEK	2	2185.10	2185.11
**20.**	DAIPENLPPLTADFAEDK	2	2244.17	2244.18
	**β-Galactosidase (P00722)** [b]			
**1.**	SLNGEWR	1	1005.53	1005.54
**2.**	YWQAFR	1	1014.53	1014.54
**3.**	FNDDFSR	1	1044.49	1044.50
**4.**	QNNFNAVR	1	1106.59	1106.60
**5.**	WLPAMSER	1	1133.59	1133.60
**6.**	WVGYGQDSR	1	1211.60	1211.61
**7.**	GDFQFNISR	1	1227.63	1227.64
**8.**	IDPNAWVER	1	1243.66	1243.67
**9.**	LAAHPPFASWR	1	1396.76	1396.77
**10.**	HQQQFFQFR	1	1409.72	1409.73
**11.**	ELNYGPHQWR	1	1443.73	1443.74
**12.**	LWSAEIPNLYR	1	1505.83	1505.84
**13.**	LPSEFDLSAFLR	1	1538.84	1538.85
**14.**	DWENPGVTQLNR	1	1572.79	1572.80
**15.**	QFCMNGLVFADR	1	1590.74	1590.75
**16.**	APLDNDIGVSEATR	1	1601.83	1601.84
**17.**	LSGQTIEVTSEYLFR	1	1887.00	1887.01
**18.**	VNWLGLGPQENYPDR	1	1901.97	1901.98

**19.**	WSDGSYLEDQDMWR	1	1931.84	1931.85
**20.**	IENGLLLLNGKPLLIR	2	2064.32	2064.34
**21.**	DVSLLHKPTTQISDFHVATR	2	2553.41	2553.43
**22.**	QLIELPELPQPESAGQLWLTVR	1	2661.48	2661.50
**23.**	VVQPNATAWSEAGHISAWQQWR	1	2666.34	2666.36
	**α-Lactalbumin (P00711)** [b]			
**1.**	VGINYWLAHK	2	1488.86	1488.87
**2.**	FLDDDLTDDIMCVK	2	1976.93	1976.95
	**β-Lactalbumin (P02754)** [b]			
**1.**	IDALNENK	2	1204.68	1204.69
**2.**	LIVTQTMK	2	1221.76	1221.77
**3.**	VLVLDTDYK	2	1353.79	1353.80
**4.**	VAGTWYSLAMAASDISLLDAQSAPLR	1	2851.48	2851.50
	**Lysozyme (P00698)** [b]			
**1.**	HGLDNYR	1	1018.52	1018,.53
**2.**	GTDVQAWIR	1	1189.65	1189.66
**3.**	FESNFNTQATNR	1	1572.76	1572.77
**4.**	NTDGSTDYGILQINSR	1	1897.94	1897.95
	**Apotransferrin (P02787)** [b]			
**1.**	APNHAVVTR	1	1108.64	1108.65
**2.**	ASYLDCIR	1	1130.55	1130.56
**3.**	DSAHGFLK	2	1162.65	1162.66
**4.**	DGAGDVAFVK	2	1266.70	1266.71
**5.**	WCALSHHER	1	1328.61	1328.62
**6.**	EGYYGYTGAFR	1	1427.67	1427.68
**7.**	DYELLCLDGTR	1	1487.70	1487.71
**8.**	SASDLTWDNLK	2	1537.82	1537.83
**9.**	EFQLFSSPHGK	2	1564.84	1564.85
**10.**	MYLGYEYVTAIR	1	1622.84	1622.85
**11.**	DQYELLCLDNTR	1	1672.78	1672.80
**12.**	CGLVPVLAENYNK	2	1753.93	1753.94
**13.**	DCHLAQVPSHTVVAR	1	1822.92	1822.934
**14.**	TAGWNIPMGLLYNK	2	1866.03	1866.04
**15.**	LCMGSGLNLCEPNNK	2	1972.91	1972.92
**16.**	SAGWNIPIGLLYCDLPEPR	1	2304.17	2304.18
**17.**	EDLIWELLNQAQEHFGK	2	2358.24	2358.26
**18.**	QQQHLFGSNVTDCSGNFCLFR	1	2637.16	2637.18

[a]Amino-acid sequences of peptides identified after tryptic digestion and iTRAQ labelling on the basis of their CID spectrum. [b]According to "UniProtKB" (http://www.uniprot.org/) and Applied Biosystems iTRAQ™ Reagents - Chemistry Reference Guide. [c]All mass values are listed as monoisotopic mass.

Table 2S: Identified proteins and peptide sequences from Tumoral and Non Tumoral prostate tissue by MS/MS Data Processing

	Protein ID	Protein Name	m/z	N° of labels	Modifications
**1**	**P63104**	**14-3-3 protein zeta/delta**			
		DSTLIMQLLR	1333.76	1	
		TAFDEAIAELDTLSEESYK	2276.09	1	
		YLAEVAAGDDK	1295.66	1	
**2**	**Q9P2A4**	**ABI gene family member 3**			
		SIKAPATPASATLGRPPR	1935.13	1	
		LSAASSAFSLASAGSAEGVGGAPTPK	2435.25	1	
		DNELSFSEGTVICVTR	1913.94	1	
**3**	**P68032**	**Actin, alpha cardiac muscle 1**			
		HQGVMVGMGQK	1315.67	1	
		SYELPDGQVITIGNER	1934.99	1	
		AGFAGDDAPR	976.45	----	
		AGFAGDDAPR	1120.55	1	
		ALPHAIMR	1052.62	1	
		AVFPSIVGR	945.55	----	
		AVFPSIVGR	1089.65	1	
		AVFPSIVGRPR	1198.71	----	
		AVFPSIVGRPR	1342.81	1	
		EITALAPSTMK	1287.71	2	Glu->pyro-Glu@N-term
		GILTLK	932.64	2	
		GYSFVTTAER	1130.55	----	
		GYSFVTTAER	1274.65	1	
		HQGVMVGMGQK	1458.77	1	Diphthamide(H)@1 Deamidated(Q)@2
		HQGVMVGMGQK	1459.78	2	
		IIAPPERK	1067.67	1	
		KAGFAGDDAPR	1120.54	----	Oxidation(D)@7
		KAGFAGDDAPR	1120.54	1	Oxidation(F)@4
		MTQIMFETFNVPAMYVAIQAVLSLYASGR	3251.63	----	
		RGILTLK	944.64	1	iTRAQ4plex(K)@7
		SGGTTMYPGIADR	1325.62	----	
		SYELPDGQVITIGNER	1790.89	----	
		TTALVCDNGSGLVK	1522.79	1	Deamidated(N)@8
**4**	**P25054**	**Adenomatous polyposis protein**			
		SSADSTSARPSQIPTPVNNNTK	2416.22	1	
		VTPFNYNPSPRK	1563.84	1	
		TDSTESSGTQSPK	1468.69	1	
		QNVGNGSVPMRTVGLENR	1927.98	----	
**5**	**O95996**	**Adenomatous polyposis coli protein 2**			
		GGKEAEK	1006.58	2	
		AGTEAGPGARGGR	1156.58	----	
		DPLNLALR	911.53	----	
		GGGAGGAGLHF	900.43	----	
		MTRDPLNLALR	1300.70	----	Deamidated(N)@7
		PEKRGAASVK	1186.70	----	
		RPEKRGAASVK	1342.80	1	
		RRRKPR	1012.67	1	
		SAEQSRGAGK	1134.60	1	
**6**	**Q08462**	**Adenylate cyclase**			
		KQWLK	1134.71	3	
		EKIK	949.68	2	
		FSGVEK	954.73	1	
		GIINVK	932.68	1	Deamidated(N)@4
		QNEYYCR	1120.65	1	Deamidated(N)@2
		SLSQSNVA	1029.40	1	Phospho(S)@5
		TKSQ	911.55	1	Phospho(T)@1; Phospho(S)@3
**7**	**P51825**	**AF4/FMR2 family member 1**			
		GSRSSSADK	1038.53	1	
		AGSRTSLQGGR	1089.58	----	
		TLNKHFESSSK	1421.75	1	
		SFSDATAPTQEKIFAVLCMR	2359.19	1	
		SAYSVYSETVDLIK	1718.89	1	
**8**	**P10696**	**Alkaline phosphatase, placental-like**			

		AGKSVGVVTTTR	1175.67	----	
		GNEVISVMNR	1134.56	----	Oxidation(M)@8
		KAYTVLLYGNGPGYVLK	2000.13	1	
		GSSIFGLAPGK	1177.67	1	
		NLVQEWLAK	1244.71	1	
		HQGARYVWNR	1287.63	----	Deamidated(Q)@2
		LQPAQTAAK	927.53	----	
**9**	**Q99490**	**Arf-GAP with GTPase, ANK repeat and PH domain-containing protein 2**			
		RTSLFANRR	1120.63	----	
		DPGSPRGAEEPGK	1440.72	1	
		ELLGAELR	900.51	----	
		KQQQLLAACK	1274.74	1	
		VFQEVAQK	1092,61	1	
		RTSLFANRR	1120.63	----	
		AKPSRDSSR	1147.63	1	
**10**	**Q8TF01**	**Arginine/serine-rich protein PNISR**			
		SRSPTIK	932.56	1	
		ARVKIRDRR	1169.73	----	
		EEKDFK	939.49	1	
		EYQMMLLTK	1300.68	1	
		KEKEK	949.59	2	
		KEKK	964.65	3	
		KEKQK	949.59	2	Deamidated(Q)@4
		KKEK	964.65	3	
		KKPK	932.66	3	
		QMEEEK	937.44	1	
		RSRSRDR	932.51	----	
		SESPGSSK	922.46	1	
		SIDKDRKK	1421.88	3	
		SRSPTIK	933.55	1	Deamidated(R)@2
		SRSRDRR	932.51	----	
**11**	**O14525**	**Astrotactin 1**			
		HASGPGLK	910.52	1	
		NFRCISDRK	1282.68	1	
		RRSRVGSPR	1070.63	----	
		RSSLKYLGCR	1182.64	----	
		SSLKYLGCR	1170.64	1	
		TLWGVDNTGRR	1274.66	1	
**12**	**Q4LE39**	**AT-rich interactive domain-containing protein 4B**			
		ACTGQKRVK	1134.65	1	
		ACTGQKRVK	1134.65	1	
		CAYKK	900.52	2	
		IDHLTNNR	983.49	----	Deamidated(N)@7
		KENIK	919.58	2	
		LLNNSDER	960.47	----	
		NKLDK	906.55	2	Deamidated(N)@1
		SPARTQSPGK	1172.65	1	
		VKDAQGGGSSSK	1120.56	----	
**13**	**O75815**	**Breast cancer anti-estrogen resistance protein 3**			
		EKLK	949.64	3	
		QNEAPGPR	1012.63	1	
		EQNLPR	900.54	1	
		QNEAPGPR	1012.58	1	
**14**	**Q9UIF8**	**Bromodomain adjacent to zinc finger domain protein 2B**			
		EREKLK	1090.68	2	
		AESVQIK	918.54	1	
		ASGQTLKIK	1089.67	1	
		CNQEQSK	980.46	1	
		GIREKALQK	1186.74	1	
		GQTKSTSSGGGNR	1237.58	----	Deamidated(N)@12
		IKEKEMRR	1089.62	----	
		KKPK	932.66	3	
		KQQEK	949.56	2	Deamidated(Q)@3
		NKDLK	906.55	2	Deamidated(N)@1
		QKEQIK	918.54	1	Deamidated(Q)@1
		QKEQIK	918.54	1	Deamidated(Q)@4

		SVMFGQDR	939.43	----	
		TGEDESSAHALERK	1673.82	1	
		VYCQICRK	1156.61	1	
**15**	**Q9NYQ7**	**Cadherin EGF LAG seven-pass G-type receptor 3**			
		MQVEPR	903.62	1	
		TAPEPAPK	939.67	1	Glu->Asn@4
		AATLGHR	949.61	1	Phospho(T)@3
		FRRAANR	1034.66	1	
		GLLSVTVTR	1089.77	1	
		LAQRLR	900.54	1	
		MQVEPRNVDR	1387.91	1	
		VTSANRAR	1098.44	1	Phospho(S)@3
**16**	**O15484**	**Calpain-5**			
		GGLISASIK	989.61	1	
		GGGCINHK	929.47	1	
		RKRPK	972.67	2	
		DEFLGQVHLK	1329.73	1	
**17**	**Q66K79**	**Carboxypeptidase Z precursor**			
		LLGCAVLAPR	1012.60	---	
		NPAATCVDLQLR	1300.67	---	
		MFSPTPDEK	1195.58	1	
		GVVTDKFGKPVK	1418.85	1	
		VIIPARMK	1071.68	1	
**18**	**P35222**	**Catenin beta-1**			
		MVALLNK	932.57	1	
		AHQDTQRR	1011.51	----	
		AHQDTQRR	1012.49		Deamidated(Q)@3
		AIPELTK	915.56	1	
		AVRLAGGLQK	1156.73	1	
		MAVRLAGGLQK	1287.77	1	
		EASRHAIMR	1052.54	----	Glu->pyro-Glu@N-term
		NEGVATYAAAVLFR	1481.77	----	
		RLVQLLVR	996.67	----	
**19**	**Q96P48**	**Centaurin-delta-2**			
		LRPPTCWGFTVVHETEK	2144.11	1	
		SVAAFTADPLSLLR	1460.81	----	
		VYLGVK	822.52	1	
		YFILNSSCLRLYK	1763.96	1	
**20**	**Q9HC77**	**Centromere protein J**			
		SLSPSGLK	932.55	1	
		DRASEFK	996.52	1	
		KLQKER	1089.70	2	
		KQEQLK	918.54	1	Deamidated(Q)@2
		KTALKNK	1090.72	2	
		NYLPMQGNPPR	1287.62	1	Deamidated(N)@8
		QKQLK	932.61	2	
		RQQLEQLQR	1198.67	----	
		RSKSAPPR	1042.62	1	
		VQRDGNK	960.53	1	
		VTEQIQEANDK	1274.62	----	
		SGRIRVK	959.62	1	
**21**	**O14647**	**Chromodomain-helicase-DNA-binding protein 2**			
		RHMDAHR	938.44	----	Oxidation(M)@3
		DAELVDK	933.50	1	
		KDVEK	906.55	2	
		KKEK	964.65	3	
		KKPK	932.66	3	
		QKKVK	918.63	2	
		RHMDAHR	922.44	----	
**22**	**Q8TD26**	**Chromodomain-helicase-DNA-binding protein 6**			
		KAKEHK	1172.75	3	
		AKEHK	900.55	2	
		ASLKLGLDK	1088.68	1	
		DEKIK	920.57	2	
		DREPKPKR	1313.79	2	
		DTGPRRRGR	1070.59	----	
		GGTNGVQQLSK	1089.55	----	Deamidated(N)@4
		GSKDREPK	1060.59	1	

		KKEK	964.65	3	
		KKKTK	1064.75	3	
		LDNICHVVLK	1298.72	1	Deamidated(N)@3
		MKIQKK	1064.67	2	Deamidated(Q)@4
		MKIQKK	1079.68	2	Oxidation(M)@1
		NYAQHK	905.46	1	Deamidated(N)@1
		PTGEEVK	903.49	1	
		QKKEK	949.59	2	Deamidated(Q)@1
		RRGRRK	972.64	1	
		RRRRGRR	1012.65	----	
		RSGRQVKR	1130.70	1	
		TKSRK	907.59	2	
		VGMPDEK	919.47	1	
		YYRAILEK	1055.59	----	
**23**	**Q02388**	**Collagen alpha 1(VII)**			
		EGPRGPK	1028.69	2	
		AQHRER	940.61	1	
		DGVPGIRGEK	1315.75	2	
		EGPRGPK	1028.67	2	
		GDSAVILGPPGPR	1379.85	1	
		GEPGEMGLR	1089.71	1	
		GPRGLK	915.53	2	
		GSSGPK	900.59	2	Phospho(S)@3
**24**	**P08123**	**Collagen alpha 2(I) chain**			
		TGHPGTVGPAGIR	1363.75	1	
		GPRGLPGLK	1038.65	1	
		GETGPSGPVGPAGAVGPRGPSGPQGIR	2412.24	----	
		DGNPGNDGPPGRDGQPGHK	2015.93	1	
**25**	**P08572**	**Collagen alpha 2(IV) chain**			
		EGFPGPPGFIGPR	1471.97	1	
		GRPGLK	915.53	2	
		GEAGFFGIPGLK	1336.74	1	
		ATPFIECNGGR	1308.65	1	
**26**	**P12277**	**Creatine kinase B-type**			
		GGNMKEVFTR	1282.67	1	
		VISMQKGGNMK	1336.72	1	
		LPNLGKHEK	1179.69	1	
		AIEKLAVEALSSLDGDLAGR	2028.10	----	
**27**	**Q9P0U4**	**CXXC-type zinc finger protein 1**			
		HYCWEKLR	1134.55	----	
		KIRQK	960.65	2	
		HYCWEK	1009.47	1	
		LPKR	657.45	1	
		VPADEVCGCPLVR	1501.76	1	
**28**	**Q9NZJ0**	**Denticleless protein homolog**			
		KAENPSPR	1042.58	1	
		CNKKDGFYR	1274.64	1	
		ICTYFHR	939.45	----	
		KNYTAYR	1060.55	1	Deamidated(N)@2
		KPKK	932.66	3	
		NRVKRR	972.63	1	
		RKAENPSPR	1198.68	1	
		TSPAKARSPINR	1441.84	1	
		VWDLRK	960.57	1	
**29**	**P17661**	**Desmin**			
		KVHEEEIR	1327.75	2	
		AQYETIAAK	1282.72	2	
		KLLEGEESR	1348.85	2	
		HLKDEMAR	1287.70	2	
		QELNDR	918.47	1	
		EEIQLK	903.52	1	
		EIQLK	918.58	2	
		LEEEIR	932.51	1	
**30**	**Q08554**	**Desmocollin – 1A/1B precursor**			
		DTGDIFCTRSIDR	1498.70	----	
		DTALKRTK	932.55	----	
		EQQEIK	919.49	1	Deamidated(Q)@3
		GGGHQTLESVK	1256.67	1	
		GGYTLDSNK	1098.55	1	

**31**	**Q14117**	**Dihydropyrimidinase**			
		DRTCTPTPVER	1274.61	----	
		KPFAEYIYKR	1458.82	1	
		GKVVYEAGVFSVTAGDGK	1928.02	1	
		EDATAGTR	964.48	1	
**32**	**Q9Y485**	**DmX-like 1 protein**			
		SSNESTLSK	952.46	----	
		SILRKK	1032.71	2	
		KAINNVR	958.59	1	
		ILSPFSQK	1064.61	1	Deamidated(Q)@7
		HQLLISGGR	980.56	----	
		ENFQEK	938.47	1	
**33**	**Q9NPF5**	**DNA methyltransferase 1-associated protein**			
		KAPKKK	1275.90	4	
		FPDFKSAGVTLR	1337.72	----	
		MFFHWR	939.43	----	Oxidation(M)@1
		LPSSVGQK	959.56	1	
		RSVEDLK	990.57	1	
		WMPFTNPAR	1120.52	----	Deamidated(N)@6
		RESASSSSSVK	1268.65	1	
**34**	**Q92878**	**DNA repair protein RAD50**			
		NIDQCSEIVK	1292.67	1	
		SEYVEKFYR	1364.69	1	
		NFQLLVITHDEDFVELLGRSEYVEK	3137.62	1	
		NFQLLVITHDEDFVELLGR	2258.18	----	
		NFQLLVITHDEDFVELLGR	2402.28	1	
**35**	**O60870**	**DNA/RNA-binding protein KIN17**			
		QDLDDEEK	1135.52	1	
		FIEEQVR	920.48	----	
		HKKK	972.67	3	
		KEKK	964.65	3	
		TIGSSASVK	993.57	1	
**36**	**Q8TD84**	**Down syndrome cell adhesion molecule-like protein 1**			
		SAQGEGDDVKK	1421.74	2	
		SEAFFRK	1028.56	1	
		SAHSTRNR	928.47	----	
		VQLLIEDK	1101.66	1	
		HLTLDPASK	1125.64	1	
**37**	**Q96DT5**	**Dynein heavy chain 11, axonemal**			
		QDHYDWGLR	1189.54	----	
		GWARCVLPPRR	1342.72	----	Dioxidation(C)@5
		KKLVDLDR	1274.80	2	
		KIELK	918.62	2	
		QRECEADLLK	1348.70	1	
		LEKLK	918.62	2	
		NLLKK	903.62	2	
		ELEAKKIR	1274.80	2	
		DRSWKAAK	1249.72	2	
		IGLQTEK	932.55	1	
		MLETAYK	999.53	1	
		LVNGIQKLK	1300.85	2	
		RMLETAYK	1155.63	1	
		ANPSLDTWK	1175.62	1	
		NTIKKGK	932.60	1	
		YLLMLKK	1052.66	1	
		IGLQTEKVSR	1130.65	----	
		RMLETAYK	1011.53	----	
		LASYFMGR	960.46		Oxidation(M)@6
**38**	**Q8WXX0**	**Dynein heavy chain 7, axonemal**			
		KDPREK	1060.63	2	
		RFTAVDK	980.56	1	
		AFASSVTPDR	1050.52	----	
		EFIMGLFDR	1127.55	----	
		QAALKEVQDK	1237.72	1	
		SVLTAAGNLKLK	1358.85	1	
**39**	**Q03001**	**Dystonin**			
		NTGHLHPTPR	1130.57	----	Deamidated(N)@1
		AVLRIADER	1042.60	----	

		DSVICEDK	1052.50	1	
		EAAENELR	932.43	----	Deamidated(N)@5
		EKGRMR	920.52	1	
		FYVAEK	900.49	1	
		KDQAEK	1006.58	2	
		KEKQK	949.59	2	Deamidated(Q)@4
		NTGHLHPTPR	1130.57	----	Deamidated(N)@1
		NTNIARK	960.57	1	
		PHHDRRGFPR	1274.66	----	
		QALYYSELLR	1238.64	----	Gln->pyro-Glu@N-term
		RRVAAAAAAR	1012.61	----	
**40**	**P14625**	**Endoplasmin**			
		ETLQQHK	1172.81	2	Deamidated(Q)@5
		ALKDK	1006.62	3	
		DKIEK	1064.72	3	
		EEEEKK	1079.73	2	
		EEEEKK	935.63	1	
		EELTVK	1006.64	2	
		EKSEK	1052.72	3	
		ETLQQHK	1172.77	2	Deamidated(Q)@5
		ETLQQHK	1028.67	1	Deamidated(Q)@5
		KIEK	949.68	3	
		TLDMIK	1088.72	3	Phospho(T)@1;
**41**	**Q96J88**	**epithelial stromal interaction protein 1**			
		VNNAFLDR	1092.59	1	
		QQQEQER	945.44	----	
		SELLELKR	1131.68	1	
		AKIHQTEHR	1263.70	1	
**42**	**Q8TAM0**	**G protein-coupled receptor 62**			
		GSRLRSDSLDSR	1348.69	----	
		GSRLRSDSLDSR	1462.79	1	
		AALRPPRPARGSRLR	1674.01	----	
		AALRPPRPARGSRLR	1818.11	1	
		ACTPQAWHPR	1310.65	1	
**43**	**O94808**	**Glucosamine--fructose-6-phosphate aminotransferase [isomerizing] 2**			
		CGIFAYMNYR	1237.55	----	
		DDTESSK	925.42	1	
		TCTLENVK	1052.54	1	Deamidated(N)@6
		NLAKSVTVE	1104.64	1	
		DPCFAKCQNALQQVTAR	1892.91	----	
**44**	**Q6PCE3**	**Glucose 1,6-bisphoshate syntase**			
		LIDALIENFLQPSK	1600.89	----	
		LIDALIENFLQPSK	174499	1	
		TSGTEPKIK	1104.63	1	
		DVTTGYDSSQPNK	1555.73	1	
**45**	**P30711**	**Glutathione S-transferase theta 1**			
		LMPWVLAMIR	1373.79	1	
		DFPPADPTIK	1244.66	1	
		LATWRQR	930.53	----	
		DFPPADPTIKQK	1644.91	2	
**46**	**Q9NU53**	**Glycoprotein integral membrane protein 1**			
		INVTTLK	932.59	1	
		VDVIPVTAINLYPDGPEK	2084.13	1	
		VFFPVSEYK	1259.68	1	
		GILQLDK	930.58	1	
**47**	**Q14789**	**Golgin subfamily B member 1**			
		MVQLNEEK	1135.58	1	Deamidated(N)@5
		AKQQIQRK	1287.81	2	
		AMSSLQNDR	1021.47	----	
		EEEIRLK	1060.61	1	
		EEKLK	934.58	2	
		EMEIEK	922.47	1	
		KEKK	964.65	3	
		KLEEK	934.58	2	
		LKTIK	932.64	2	Acetyl@N-term
		LSALFSSSQK	1211.68	1	
		SMSSLQDDR	1038.45	----	
		SMSSLQNDR	1038.45		Deamidated(N)@7

**48**	**Q99062**	**Granulocyte colony stimulating factor receptor**			
		QRQLDPR	1056.70	1	
		KENIR	947.62	2	
		LEPPMLR	999.41	1	
		SFKSR	916.58	2	Phospho(S)@1; Phospho(S)@4
		LTVLEEDEK	1219.65	1	
**49**	**Q03113**	**Guanine nucleotide-binding protein subunit alpha-12**			
		GSRVLVDAR	972.56	----	
		IIHGREFDQK	1386.76	1	
		DTILQENLK	1217.68	1	
		YLVQCFDR	123710	1	
		YLVQCFDRK	1315.69	1	
**50**	**Q96LI6**	**Heat shock transcription factor, Y-linked**			
		ESSVLSKLK	1134.69	----	
		LALQPSPLDK	1225.72	1	
		SAAPHSR	906.10	1	
		YPLVSVNEAPYR	1407.73	----	
		NLNMPLTR	1102.61	1	
**51**	**P69905**	**Hemoglobin subunit alpha**			
		GHGKK	958.69	3	
		VGAHAGEYGAEALER	1673.92	1	
		VLTSK	915.53	2	Phospho(S)@4
		VDPVNFK	962.54	1	
		LRVDPVNFK	1231.72	1	
**52**	**P68871**	**Hemoglobin subunit Beta**			
		VVAGVANALAHKYH	1593.89	1	
		VVAGVANALAHKYH	1737.99	2	
		EFTPPVQAAYQK	1522.80	1	
		LHVDPENFR	1270.66	1	
**53**	**P09105**	**Hemoglobin subunit theta-1**			
		FLSHVISALVSEYR	1764.97	1	
		HYPGDFSPALQASLDK	1889.95	1	
		VADALSLAVER	1257.74	1	
**54**	**Q8TEK3**	**Histone-lysine N-methyltransferase, H3 lysine-79 specific**			
		HSPLTASAR	939.50	----	
		KPKK	932.66	3	
		RRGRRK	972.64	1	
		LTSLPHK	939.57	1	
		EAGEGGLPLCGPTDK	1443.68	----	
		TPLLSGKAAK	1129.70	1	
		TPLLSGKAAK	1273.81	2	
**55**	**P17482**	**Homeobox protein Hox-B9**			
		RMKMKK	1253.79	3	
		DRRHEVAR	1038.55	----	
		DRRHEVAR	1182.66	1	
		SANWLHAR	954.49	----	
		QVKIWFQNR	1362.77	1	
		LLNLSER	988.59	1	
**56**	**Q9HAS2**	**Homeodomain-interacting protein kinase 3**			
		GTNEIVAIK	1088.64	1	
		FSPLPLK	945.59	1	
		GTFGQVVK	979.57	1	
		ILKNHPSYAR	1198.67	----	
		KKNK	949.65	3	
		NKQCQNR	1034.53	1	
		PENIMLVDPVR	1282.68	----	
**57**	**P42858**	**Huntingtin**			
		KELSATK	1064.66	2	
		GATAILCGTLICSILSRSR	1935.05	----	
		SPIRRK	900.59	1	
		HQIEEELDRR	1468.76	1	
		LEQVDVNLFCLVATDFYR	2145.07	----	
		KGFPCEAR	907.44	1	
**58**	**Q9Y4L1**	**Hypoxia up-regulated protein 1**			
		TLGGLEMELR	1134.58	----	Oxidation(M)@7
		EKKQK	949.59	2	Deamidated(Q)@4

		LAGLFNEQRK	1175.65	----	
		LCQGLFFR	949.53	----	Cys->Dha(C)@2
		NATLAEQAK	1089.60	1	
		VFGSQNLTTVK	1337.75	1	
**59**	**P23677**	**Inositol 1,4,5-trisphosphate 3-kinase A**			
		RGGQVPNGLQR	1182.63	----	Deamidated(Q)@10
		RGGQVPNGLQR	1181.65	----	
		APRRSVGELR	1182.67	----	Acetyl@N-term
		ARERPK	900.55	1	
		GGQVPNGLQR	1025.55	----	
		KDMYK	972.54	2	
		AGVWLIDFGK	1249.70	1	
**60**	**O15503**	**Insulin-induced protein 1**			
		REWASVMR	1034.52	----	
		ASAAGLAAK	903.53	1	
		EWASVMR	1022.52	1	
		CIAVFVGINHASAK	1573.86	1	
**61**	**P24593**	**Insulin-like growth factor binding protein 5 precursor**			
		QESEQGPCRR	1189.54	----	
		ASLQELK	932.55	1	
		KKLTQSK	1120.73	2	
**62**	**Q9BR39**	**Junctophilin 2**			
		EAAARAPK	939.55	1	Glu->pyro-Glu@N-term
		RMLQLK	932.58	1	
		ETPAKLEPKPIIPK	1705.03	1	
		GPEPARETPAK	1152.60	----	
**63**	**Q01546**	**Keratin, type II cytoskeletal 2 oral**			
		SGGAGGGACGFR	996.43	----	
		KLLEGEECR	1220.64	1	
		DYQELMNVK	1283.64	1	
		AKDDLAR	932.53	1	
**64**	**Q96L93**	**Kinesin-like protein KIF-16B**			
		HTICEFSPFFK	1499.74	1	
		HTICEFSPFFKK	1627.84	1	
		HTICEFSPFFKK	1771.94	2	
		LFGNKDER	1122.60	1	
		YAELAALEFPPKK	1476.81	----	
		YAELAALEFPPKK	1620.91	1	
**65**	**Q8N4N8**	**Kinesin-like protein KIF2B**			
		RALDVNTR	944.53	----	
		DTTISGK	865.47	1	
		IPYVQSEEQK	1364.71	1	
		GHYPIGHEAPR	1377.71	1	
**66**	**Q32MZ4**	**Leucine-rich repeat flightless-interacting protein 1**			
		EEEQVK	905.47	1	
		AMVSNAQLDNEK	1463.73	1	
		EEEQVK	905.47	1	
		EELNALK	960.55	1	
		EEMLEK	922.47	1	
		KKNK	949.65	3	
**67**	**Q9UNZ5**	**Leydig cell tumor 10 kDa protein homolog**			
		GGRVIAPK	941.60	1	
		KGAAAATSSK	1035.59	1	
		KIEHDVVMK	1242.70	1	
**68**	**Q9H2C1**	**LIM/homeobox protein Lhx5**			
		AAFAATPKPTR	1130.63	----	
		EGSLNSVSSCTDR	1354.59	----	
		HAFFRSPR	1161.64	1	
		QLSALGAR	959.57	1	
		GPRTTIK	916.57	1	
**69**	**O75334**	**Liprin-alpha2**			
		QMEEKNR	934.44	----	
		KAPKKK	1275.90	4	
		KGIKSSIGR	1089.69	1	
		KHRRK	1012.67	2	
		KKEK	964.65	3	
		QNYEMAQMK	1287.58	1	Deamidated(N)@2

		RLDESDDK	1121.55	1	
**70**	**Q9NZR2**	**Low-density lipoprotein receptor-related protein 1B**			
		RDGRNPTILR	1198.66	----	Deamidated(N)@5
		DKSDEK	1009.54	2	
		NCETSCSK	1015.43	1	
		NELFLFYGK	1274.69	1	
**71**	**Q9H239**	**Matrix metalloproteinase-28**			
		YWRLDQAK	1223.66	1	
		LILFKGAR	1061.69	1	
		RPETQGPK	1056.59	1	
**72**	**Q9NR99**	**Matrix-remodeling-associated protein 5**			
		NILGSDSK	977.54	1	
		DAGFYK	844.43	1	
		LNCMAMGIPKADITWELPDK	2390.20	1	
		VSLLDNGTLTVR	1287.73	----	
		VGLKPEANK	1099.66	1	
**73**	**Q96JG8**	**Melanoma-associated antigen D4**			
		FIAQNQNR	1134.61	1	
		ARHETSK	972.53	1	
		ASEAVLWEALRK	1372.76	----	
		LITDDFVKQK	1350.77	1	
**74**	**Q8NFU7**	**Methylcytosine dioxygenase TET1**			
		LSLVFYQHK	1278.73	1	
		RELHATTPVEHPNR	1656.86	----	
		FEAKEAK	966.53	1	
		SETEPHFILK	1344.72	1	
		AAMMTEVLAHKIR	1614.89	1	
		TCFTQPVPR	1048.52	----	
**75**	**P11137**	**Microtubule-associated protein 2**			
		SSLEKHRK	984.56	----	
		AGKSGTSTP	949.51	1	
		ARVDHGAEIITQSPGR	1706.89	----	
		ASGLNIDDR	960.47	----	
		CGSLKNIR	1034.59	1	
		DFKEK	954.55	2	
		EEEQIEK	1048.53	1	
		EFDQEK	939.45	1	
		EHAKK	900.55	2	
		EPSTVSRDEVR	1274.63	----	
		GGRGEGLVR	900.50	----	
		KEKPFK	1064.67	2	
		KLEEK	934.58	2	
		MFTIDPK	1011.53	1	Oxidation(M)@1
		NIRHRPGGGR	1120.61	----	Deamidated(N)@1
		SSVASPRRL	972.56	----	
**76**	**Q9NU22**	**Midasin**			
		QFFDRVQAK	1282.70	1	
		DTSPVFQR	949.47	----	
		GSCVPHPSR	939.45	----	
		KELSPALRNR	1327.79	1	
		KKLQK	932.65	2	
		LTEYQK	925.51	1	
		MCLTFMK	1033.50	1	Oxidation(M)@1
		MRTEEDK	1052.52	1	
		QFFDRVQAK	1282.70	1	
		QGGGRKGLPR	1169.70	1	
		QLEMWQPR	1070.51	----	Gln->pyro-Glu@N-term
		RHTEKSTK	1274.74	2	
		RNLPPGIR	922.55	----	
		SGGKELEGKGQK	1361.75	1	
		TTAMNAQRLLR	1274.70	----	
		VLNKVLAIR	1169.78	1	
		LKTIK	932.64	2	Acetyl@N-term
**77**	**P08235**	**Mineralocorticoid receptor (MR)**			
		RNDCIIDK	1120.59	1	
		NDCIIDKIR	1089.57	----	
		PENVSSSTLR	1089.55	----	
		PSTLSCVNTPLR	1287.67	----	

		QMIQVVK	989.59	1	
		RNDCIIDK	976.49	----	
**78**	**O60336**	**Mitogen-activated protein kinase-binding protein 1**			
		RLLDKWVELR	1327.78	----	
		GGKQQGPSSPQR	1370.72	1	
		GSQGEDGTLIK	1249.63	1	Deamidated(Q)@3
		IKNLLR	900.61	1	
		MPSAEQSR	905.41	----	
		NIVVASNKVSSR	1274.71	----	Deamidated(N)@7
		QRLAELRQR	1169.69	----	
		SQPCSYPHIIR	1300.65	----	
**79**	**Q8WV50**	**Mitotic checkpoint serine/threonine-protein kinase BUB1 beta**			
		ALWKVGK	945.60	1	
		DGELWNK	1005.51	1	
		TVQILEGQK	1015.58	----	
		IHDPYDCNK	1248.58	1	
**80**	**P02686**	**Myelin basic protein**			
		GVDAQGTLSK	1120.60	1	Deamidated(Q)@5
		GESEKK	965.55	2	
		IFKLGGR	934.59	1	
		LGGRDSR	984.49	1	Phospho(S)@6
**81**	**P60600**	**Myosin light polypeptide 6**			
		VLGNPK	915.59	2	
		EAFQLFDR	1025.51	----	
		EGNGTVMGAEIR	1249.58	----	Oxidation(M)@7
		LRVFDK	921.56	1	
		SGDAGAAVLR	1060.58	1	
**82**	**P35749**	**Myosin-11**			
		VTAEAKIKK	1275.82	2	
		AARNKAEK	1175.71	2	
		AKANLDK	903.54	1	
		CIIPNHEKR	1219.70	1	Cys->Dha(C)@1
		DADFNGTK	1011.49	1	
		DADFNGTKASE	1298.60	1	
		EAEAREK	976.52	1	
		EKQAATK	1064.62	2	Deamidated(Q)@3
		EQEVTVLK	1090.61	1	Deamidated(Q)@2
		GNETSFVPSRR	1249.63	----	
		HKKK	972.67	3	
		ITDVIMAFQAMCR	1498.72	----	
		IVFQEFR	939.49	----	Deamidated(Q)@4
		KLEGDLK	1090.67	2	
		LEKLK	918.62	2	
		LKKEEK	918.57	1	
		MQLAKK	1006.63	2	
		NCAAYLK	927.47	1	Deamidated(N)@1
		NTTPNFVR	949.47	----	Deamidated(N)@1
		NVHELEK	1012.55	1	
		QAATKSLK	1134.71	2	
		RGNETSFVPSR	1249.63	----	
		SLEADLMQLQEDLAAAER	2147.08	1	
**83**	**Q9UKX3**	**Myosin-13**			
		KLEGDLK	1090.67	2	
		KEKK	964.65	3	
		GGKKK	949.65	3	
		LQDLVDK	974.56	1	
		EEKLK	934.58	2	
		ACFVADNK	1011.50	1	
		GAEALKGAHK	1125.65	1	
**84**	**Q8WXH0**	**Nesprin-2**			
		KKAAIK	1090.77	3	
		CKVTHDGILAR	1356.75	1	
		DAPGTGEEAQGK	1303.62	1	

		EEIYNLK	1052.57	1	
		IWGEKEKK	1305.77	2	
		KDVEK	906.55	2	
		KEEIK	934.58	2	
		KEKK	964.65	3	
		KHLPK	910.61	2	
		KKNK	949.65	3	
		LPQLQGEIER	1182.65	----	
		NQYQMLVLK	1280.72	1	
		RSKSLK	1006.66	2	
**85**	**Q8NF91**	**Nesprin-1**			
		SMHRGSPIK	1156.64	1	
		ADGSVEEAENVMK	1522.72	1	
		DEFNLK	910.46	1	Deamidated(N)@4
		EDLEQKVASLELR	1673.92	1	
		EEKLK	934.58	2	
		EMFATMSK	1088.52	1	
		EQKEK	949.56	2	
		EVTEQEK	1006.52	1	
		FNTENLGESK	1282.64	1	
		GYQEQIASLNSK	1481.77	1	
		IEKLK	918.62	2	
		IIELKR	915.61	1	
		KEKEK	949.59	2	
		KQELK	934.58	2	Deamidated(Q)@2
		LEEQKK	918.54	----	
		LGKVNDR	945.56	1	
		LSRVESLAPEVK	1471.86	1	
		LTENAIK	932.55	1	
		LVNINSTDIADGR	1387.72	----	
		NKMNYK	941.50	1	
		QMIKIR	932.58	1	
		QMLLKSLIK	1361.88	2	
		QVKTCK	960.61	2	Cys->Dha(C)@5
		SGLNQNLTLK	1087.61	----	
		SGRGFLFR	939.52	----	
		SSVLSTGNQLLR	1274.71	----	
		TDMESTVDK	1025.45	----	
**86**	**Q9ULB1**	**Neurexin1**			
		SSNKNKK	1238.74	3	Deamidated(N)@3
		KKNK	949.65	3	
		LELDAGRVK	1144.68	1	
		SDLYIGGVAKETYK	1832.00	2	
		QGDPKMK	947.51	1	
		LAKQGDPK	1000.59	1	
**87**	**Q8NFP9**	**Neurobeachin**			
		VTYEAHK	991.53	1	
		KPEDFVR	1034.57	1	
		GKGFQHCVK	1147.61	1	
		AEKVEATEVK	1103.59	----	
		GFLVIGYLLEK	1251.73	----	
**88**	**Q6KC79**	**Nipped-B-like protein**			
		SSLKPIK	1060.70	2	
		DKVEK	906.55	2	
		ESMVKDK	980.52	1	
		GSIDQSVLK	1090.62	1	
		KEKQK	949.59	2	Deamidated(Q)@4
		KKEK	964.65	3	
		KMEMK	954.53	2	
		MNKRKR	976.60	1	
		RSLRNFR	949.53	----	Deamidated(N)@5
		RSLRNFR	949.53	----	Deamidated(R)@1
		RSLRNFR	949.53	----	Deamidated(R)@4
		SQRISQRIT	1089.60	----	Deamidated(Q)@2
		TSPNMPK	918.48	1	
**89**	**P04198**	**N-myc proto-oncogene protein**			
		EGSTPGIK	932.52	1	
		RKRAQTVAIR	1342.85	1	
		DHVPELVKNEK	1307.69	----	

		AVTTFTITVRPK	1477.88	1	
**90**	**P23497**	**Nuclear autoantigen Sp-100**			
		KAAKLK	1090.77	3	
		CIQSEDKK	1238.67	2	
		EGQEATCSR	980.41	----	
		FEDMAK	900.43	1	Oxidation(M)@4
		HKKK	972.67	3	
		KKEK	964.65	3	
		KLSTFRESFK	1386.78	1	
		RRKRGPR	925.59	----	
		VNGLQRGR	900.50	----	Deamidated(N)@2
		KTFPFLEGLR	1351.78	1	
**91**	**Q15788**	**Nuclear receptor coactivator 1**			
		RQVTSGLATRPR	1342.75	----	Deamidated(Q)@2
		QLFQEVMTR	1295.69	1	
		HTGLYCNQLSSTDLLK	1936.99	1	
		DETELAELDRALGIDK	1932.01	1	
**92**	**O00482**	**Nuclear receptor subfamily 5 group A member 2**			
		FLVLFSLDVK	1324.79	1	
		AISMQAEEYLYYK	1608.76	----	
		QVVHGK	811.49	1	
		LSTFGLMCK	1143.60	1	
		CEPDEPQVQAK	1243.56	----	
**93**	**Q5VST9**	**Obscurin**			
		EGSEATFR	1120.66	1	Phospho(S)@3
		QQMLVIK	1090.72	2	Gln->Ala@2
		DRDPK	918.61	2	
		ELLIHQLEAK	1481.86	2	
		GASPPGPQVR	1189.74	1	Phospho(S)@3
		GSETLRDGDR	1249.78	1	
		KVPK	903.62	3	
		LGSARASAELR	1274.78	1	
		LHVGITK	1055.67	2	
		QGWVSPAYLDR	1274.71	----	Gln->pyro-Glu@N-term
		TPSASPR	939.61	1	Phospho(T)@1
		VRIEAAGCMR	1249.74	1	
**94**	**Q9C0B5**	**Palmithoyltransferase ZDHHC5**			
		TTYSKSNGQPK	1354.70	1	
		DEVQLK	875.49	1	
		FGKPDGLRGR	1246.71	1	
		HIVASLQEREK	1309.72	----	
**95**	**P54317**	**Pancreatic lipase-related protein 2**			
		QYEIFK	954.51	1	Gln->pyro-Glu@N-term
		VKFLWNK	1078.65	1	
		VSVTLSGKEK	1191.70	1	
**96**	**Q8ND90**	**Paraneoplastic antigen Ma1**			
		RRLMESLR	1060.60	----	
		DNVNQAR	960.49	1	
		VVEKGAIDK	1102.66	1	
		GPAADVIR	942.55	1	
**97**	**O15018**	**PDZ domain-containing protein 2**			
		NGMSVAGNR	905.43	----	
		LERTNQLK	1145.67	1	
		GGCLAQGNCQEK	1351.62	1	
		EGHPPHSLGR	1086.54	----	
		GSDSELK	879.45	1	
**98**	**O95613**	**Pericentrin**			
		LRAQLR	900.579	1	
		DNVSLTK	1064.70	2	
		ADRSEK	993.38	2	
		AESFRK	1025.58	2	
		ALLQMVR	974.619	1	
		ANSVQK	934.60	2	
		ATPSPNSR	989.63	1	Oxidation(P)@5
		CIAGDLQK	1135.71	2	
		DDLEK	907.56	2	
		DPARGRR	971.59	1	
		ECEQPIRR	1012.63	----	Glu->pyro-Glu@N-term
		EFSFK	1025.57	2	Phospho(S)@3;

		EGANLLSMLK	1363.93	2	
		EKLK	949.68	3	
		EKLNR	947.62	2	
		ENELK	920.55	2	
		GELQSVR	1012.63	1	Phospho(S)@5
		KLEK	949.68	3	
		LELSRAVSK	1370.76	2	Phospho(S)@8;
		LEQLK	918.60	2	
		LKEK	949.58	3	
		LLGLFGETLR	1342.89	1	Phospho(T)@8
		LRAQLR	900.58	1	
		LTLMLLELR	1325.92	1	Phospho(T)@2
		NEETAQVVR	1190.69	1	Deamidated(N)@1
		NLEIDALNQR	1330.84	1	Deamidated(N)@1
		QEHQLR	954.66	1	
		QKEK	964.67	3	
		QKLK	949.62	3	Deamidated(Q)@1
		QRKTK	1172.79	3	Phospho (T)@4
		RRRK	903.62	2	
		TAVEK	915.54	2	Phospho(T)@1
		VDLVAQVK	1160.69	2	Deamidated(Q)@6
**99**	**Q5VV67**	**Peroxisome proliferator-activated receptor gamma coactivator-related protein 1**			
		KAKSPK	1090.73	3	
		SAGQSSPAK	976.52	1	
		EDFDPAPVK	1161.59	1	
		MTRSELK	978.56	1	
		YSSYRSHDHYQR	1598.71	----	
		EKPPLPATK		1	
**100**	**O00541**	**Pescadillo homolog 1**			
		EGDYVPPEK	1033.48	----	
		EALAFIIR	932.56	----	
		HKKK	972.67	3	
		KHKK	972.67	3	
		ELEAQEK	990.52	1	
		KELEAQEK	1118.61	1	
		KPRVMAGTLK	1244.76	1	
**101**	**P15259**	**Phosphoglycerate mutase**			
		GTEEAKR	934.50	1	
		AMEAVAAQGK	1135.59	1	Oxidation(M)@2
		VLIAAHGNSLR	1294.77	1	
		RVLIAAHGNSLR	1306.77	----	
**102**	**P16284**	**Platelet endothelial cell adesion molecular**			
		VILAPWKK	1098.71	1	
		NSNDPAVFK	1135.58	1	
		ANHASSVPRSK	1297.71	1	
		KDTETVYSEVR	1470.75	1	
		CESISGTLPISYQLLK	1751.92	----	
**103**	**Q9HAU0**	**Pleckstrin homology domain-containing family A member 5**			
		NKDK	792.48	2	
		QFYNK	843.44	1	
		LQQLHK	910.56	1	
		AWREYDK	1111.56	1	
		LQQLHKEK	1167.69	1	
		ELSRATAELER	1274.67	----	
**104**	**Q15149**	**Plectin**			
		INLAQK	830.52	1	
		DGKTTVK	892.52	1	
		LPVEVAYK	1062.63	1	
		LLEAAAQSTK	1031.57	1	
		QITMEELVR	1118.59	----	
		GLVDKIMVDR	1433.83	2	
**105**	**Q9NS40**	**Potassium voltage-gated channel subfamily H member 7**			
		KFEGQNK	1138.64	2	iTRAQ4plex(K)@1-7
		DRHASEDNGR	1156.51	----	
		LSFESEGEK	1025.48	----	
		ALAMKFK	952.57	1	

		IPQLTLNFSEVK	1532.88	1	
		INKFTILHYSPFK	1896.09	2	
**106**	**Q7L014**	**Probable ATP-dependent RNA helicase DDX46**			
		SWVQCGISMK	1282.64	1	
		AVVDSDKKK	1421.87	3	
		GSKSKK	1066.69	3	
		KAVVDSDKK	1421.87	3	
		KEKK	964.65	3	
		KKLQK	932.65	2	
		LNYVPLEK	1120.64	1	Deamidated(N)@2
		LWSDFK	939.51	1	
		RSRSRDR	932.51	----	
		SKKAENR	1120.67	2	
		SRSRDRR	932.51	----	
**107**	**Q8NH12**	**Probable G-protein coupled receptor 144**			
		RRDPRQGQ	1012.54	----	
		ALGVWGGAAK	1073.62	1	
		SALQRMAEK	1177.64	1	
		GGWLTATSLGHSK	1458.78	1	
		VVAVSMHPGPGMR	1337.68	----	
**108**	**P35232**	**Prohibitin**			
		NVPVITGSK	914.53	----	
		AAIISAEGDSK	1205.65	1	
		KAAIISAEGDSK	1333.74	1	
		LEAAEDIAYQLSR	1478.75	----	
		VLPSITTEILK	1357.84	1	
**109**	**P27918**	**Properdin**			
		NVTFWGRPLPR	1342.74	----	
		SGGLCQPCR	920.41	----	
		SISCQEIPGRQSRGR	1673.85	----	
		KFDGHR	903.49	1	
		HCYSIQHCPLK	1472.72	1	
		RPCLHVPACK	1267.69	1	
**110**	**Q13258**	**Prostaglandin D2 receptor**			
		NLYAMHRRLQR	1458.77	----	Deamidated(N)@1
		NLYAMHRR	1060.54	----	
		MRNLYAMHRR	1348.67	----	Deamidated(N)@3
		RNLYAMHR	1060.54	----	
		AYYGAFKDVK	1305.69	1	
		IFFHKIFIRPLR	1731.07	1	
		AYYGAFK	963.51	1	
**111**	**Q9P2B2**	**Prostaglandin F2 receptor negative regulator**			
		AQDGDFIFSK	1271.64	1	
		LENWTDASR	1091.51	----	
		QRNNSWVK	1175.64	1	
		MDVLNAFK	1081.58	1	
		NNSWVKS	1250.70	2	
**112**	**P14921**	**Protein C-ets-1**			
		DRADLNK	976.51	1	Deamidated(N)@6
		YYYDK	895.43	1	
		NIIHKTAGK	1125.68	1	
		LSDPDEVARR	1157.59	----	
**113**	**P80511**	**Protein S100-A12**			
		ELANTIK	932.55	1	
		QLLTKELANTIK	1515.92	1	
		AAHYHTHKE	1237.62	1	
**114**	**A3KN83**	**Protein strawberry notch homolog 1**			
		GVLQSLIEK	1130.69	1	
		AKNQRRR	928.55	----	
		EVNPKKK	1275.80	3	Deamidated(N)@3
		KEKK	964.65	3	
		KKNK	949.65	3	
		GEEITREAK	1176.63	1	
		GMSWEEATK	1182.56	1	
**115**	**Q13882**	**Protein-tyrosine kinase 6**			
		EEFTLCRK	1169.61	1	
		GSLLELLR	900.55	----	
		MVSRDQAHLGPK	1498.79	1	Oxidation(M)@1
		VGDFGLARLIK	1332.81	1	

**116**	**Q9Y315**	**Putative deoxyribose-phosphate aldolase**			
		VCDAVKALK	1090.64	1	
		ACGEAHLK	972.51	1	
		IGASTLLSDIER	1274.70	----	
		DSLAWLSLVK	1275.74	1	
**117**	**Q15311**	**Ra1A-binding protein 1**			
		AAKEQPK	915.54	1	
		AAKEQPKAGK	1315.79	2	
		KEKK	964.65	3	
		DLSKEER	1020.54	1	
		KKEK	964.65	3	
		DGVLEPKAAK	1171.68	1	
**118**	**Q08999**	**Retinoblastoma-like protein 2**			
		RLREINSMIR	1287.73	----	
		CLNAGSGTETAER	1274.60		Cys->Dha(C)@1
		FAEMLYYK	1208.61	1	
		KRRNSGSSDSR	1249.64	----	
		LDISDELR	960.50	----	
		LRDLCAK	962.55	1	
		QIKTFAMK	1110.64	1	
		RLREINSMIR	1287.73	----	
**119**	**Q7Z5J4**	**Retinoid acid-induced protein 1**			
		RVGKPSPK	1012.64	1	
		AAFKSGK	996.61	2	
		GNGEPATK	917.48	1	
		KKPK	932.66	3	
		LKEKVR	1060.71	2	
		LPNCRATK	1012.60	1	Cys->Dha(C)@4
		MSSPKK	965.57	2	
		PDGPADPAK	1011.52	1	
		QEEVGGVK	972.51	1	Gln->pyro-Glu@N-term
		RKRLTRGR	1042.67	----	
		RVGKPSPK	1012.64	1	
**120**	**Q5T5U3**	**Rho GTPase-activating protein 21**			
		GKSTGSLLTPTR	1361.79	1	
		DSSSEVFSDAAK	1386.65	1	
		FAILKESPR	1204.72	1	
		KKPK	932.66	3	
		LDDCPPAHTNR	1238.56	----	
		LKTLK	932.64	2	Acetyl@N-term
		LPEPLFTNDK	1317.72	1	
		TKIADRLK	1088.69	1	
		YADFIEANR	1098.52	----	
		VVDLLSNR	915.52	----	
**121**	**Q9BST9**	**Rhotekin**			
		GLTGGPKRLATK	1342.83	1	
		GLTGGPKRLATK	1342.83	1	
		GQLRTWLQSP	1185.64	----	
		GRVCISDLR	984.56	----	Cys->Dha(C)@4
		MREGACK	938.47	1	
**122**	**Q14137**	**Ribosome biogenesis protein BOP1**			
		GAGRTAAPSVR	1042.57	----	
		MHVPAPK	939.52	1	Oxidation(M)@1
		VLKGHVLTR	1166.75	1	
		TVPVGGVVK	999.63	1	
**123**	**Q9H7B2**	**Ribosome production factor 2 homolog**			
		KPKK	932.66	3	
		GGNANATVTK	1076.58	1	
		TKRAKR	903.60	1	
		GSHNKK	958.57	2	
		IYFRSYK	1120.63	1	
**124**	**Q8WV20**	**RNA binding motif, single stranded interacting protein 1**			
		KRQNPNK	1172.71	2	
		FADGGQKK	994.54	1	
		CEAVIGHFNGK	1318.67	1	
		RQNPNK	900.51	1	
**125**	**P21817**	**Ryanodine receptor 1**			
		TFSEKDK	998.52	1	

		TALTEKSK	1021.59	1	
		DVIEEQGK	1061.56	1	
		EKAQELLK	1102.66	1	
		AEAQEGELLVR	1214.6375	----	
		AQWEMPQVK	1260.65	1	
		SGPEIVKAGLR	1270.76	1	1
**126**	**O14641**	**Segment polarity protein dishevelled homolog DVL-2**			
		TGGPSRLER	972.52	----	
		KYASGLLK	1023.63	1	
		AGLIRHTVNK	1252.76	1	
		YASGLLKAGLIR	1405.86	1	
**127**	**Q99719**	**Septin-5**			
		MKDEELR	1064.55	1	
		DESGLNRK	1062.56	1	
		RMQEMLQR	1091.54	----	
		ADCLVPSEIR	1102.56	----	
		NMLIRTHMHDLK	1652.88	1	
**128**	**Q9UQ35**	**Serine/arginine repetitive matrix protein 2**			
		GSGTNGYVQR	1038.50	----	
		KKEK	964.65	3	
		KPASPK	915.59	2	
		KPIDSLR	972.60	1	
		NKSRTSQR	1120.63	1	
		NLSLVRGR	915.54	----	Deamidated(N)@1
		RGRSPSPK	1028.61	1	
		SGAGSSPETK	1064.53	1	
		SGAGSSPETK	920.43	----	
		SHGRAKRDK	1198.69	1	
		SPCPQEK	932.46	1	
		SRREKTR	932.54	----	
		SRSPQRRGRSR	1342.75	----	
		SRTPLLPR	939.57	----	
		TAAALAPASLTSAR	1300.72	----	
		TPAAAAAMNLASPR	1342.68	----	Deamidated(N)@9
		TPARRSGR	900.51	----	
		TVARTPLGQRSR	1342.76	----	Deamidated(Q)@9
**129**	**P15056**	**Serine/Threonine protein kinase B-raf**			
		GYLSPDLSK	1123.61	1	
		IGDFGLATVK	1164.67	1	
		GYLSPDLSKVR	1378.77	1	
		ALQKSPGPQR	1081.61	----	
		IGDFGLATVKSR	1407.81	1	
		IGSGSFGTVYKGK	1588.89	2	
		FEMIKLIDIAR	1492.86	1	
**130**	**Q06190**	**Serine/threonine-protein phosphatase 2A regulatory subunit B'' subunit alpha**			
		DPFAVQK	948.52	1	
		ILSASLPEK	1101.66	1	
		APMFRAAGGEK	1278.67	1	
**131**	**P42345**	**Serine/threonine-protein kinase mTOR**			
		DASAVSLSESK	1237.64	1	
		NLMEEK	907.47	1	
		RALEWLGADR	1186.63	----	
		RHNAANK	954.54	1	
		SISLSRGNNLQDTLR	1673.89	----	
		TILNLLPR	939.60	----	
		KKEK	964.65	3	
		KKKLR	960.69	2	
		RHAAVLVLR	1034.66	----	
		SEFKVYLPR	1282.73	1	
**132**	**Q96Q15**	**Serine/threonine-protein kinase SMG1**			
		RDQSTIPR	972.52	----	
		DPKTGK	933.56	2	
		HYSVTPLGTR	1130.59	----	
		ITREDDRDR	1175.58	----	
		KDEVK	906.55	2	
		KPKK	932.66	3	
		LEKIK	918.62	2	

		LEKLK	918.62	2	
		RDQSTIPR	972.52	----	
**133**	**Q15464**	**SH2 domain-containig adapter protein B**			
		VDPAVPLEK	1111.64	1	
		APGGGFKPIK	1156.67	1	
		SNQGFMHMK	1223.57	1	
		LCKECSYLVR	1357.71	1	
**134**	**Q9H1V8**	**Sodium-dependent neutral amino acid transporter SLC6A17**			
		ILGYLNTNVLSR	1363.76	----	Deamidated(N)@6
		NGSVAVVEAECEK	1478.72	1	
		FYFYMWK	1228.59	1	
		CVVENAEK	1035.52	1	
**135**	**Q96BI1**	**Solute Carrier Family 22 member 18**			
		TDAQAPLPGGPR	1179.61	----	
			1172.71	2	
		KPMPQRK	1028.61	1	
		KPMPQR	900.52	1	
		LPDVPRIFLVK	1440.90	1	
**136**	**O94956**	**Solute carrier organic anion transporter family member 2B1**			
		EARTK	748.44	1	
		KPEDSR	875.47	1	
		QSPGESTK	977.50	1	
		VLAVTDSPAR	1028.57	----	
**137**	**P11277**	**Spectrin beta chain, erythrocyte**			
		AITAATLK	932.59	1	
		DGLAFNALIHK	1342.76	1	
		FDILDQEMK	1282.65	1	
		MKVLAVEGKR	1418.88	2	
**138**	**Q9BPZ7**	**Stress-activated map kinase interacting protein 1**			
		QSAQELK	947.52	1	
		SPSHAIFK	1030.58	1	
		ADYFAQKQR	1270.66	1	
**139**	**Q15431**	**Synaptonemal complex protein 1**			
		LKEAEK	861.51	1	
		EVKDLK	875.53	1	
		AELLSVK	903.56	1	
		LQENRK	931.54	1	
		AVPSQTVSR	944.51	----	
		VIADEAVK	988.58	1	
**140**	**Q9BQ70**	**Transcription factor 25**			
		EGVRVNNR	944.49	----	Deamidated(N)@6
		HVILSEIK	1082.67	1	
		YSKPGLSMR	1038.54	----	
		SFYLALYK	1148.64	1	
		KHVLYVEHR	1324.76	1	
**141**	**Q01664**	**Transcription factor AP-4**			
		LLQQNTQLK	1229.73	1	
		RAVIVKPVR	1181.79	1	
		TLIPHTDGEK	1254.68	1	
		QELEEEQRR	1216.59	----	
**142**	**Q8NHW3**	**Transcription factor mammalian MafA**			
		GFSKEEVIR	1064.57	----	
		GGPGSAGGAGFPR	1087.53	----	
		DLYKEK	939.52	1	
		LEVGRLAK	1029.65	1	
		EPSPPQAGPGGAK	1336.69	1	
**143**	**Q8NEM7**	**Transcription factor SPT20 homolog**			
		KYLSSGR	954.55	1	
		SSLNRQQDL	1060.54	----	
		GTPTTPKF	992.55	1	
		RNVNLLEK	1129.68	1	
**144**	**P29084**	**Transcription initiation factor IIE subunit beta**			
		YNYRDKK	1275.69	2	Deamidated(N)@2
		KKKTK	1064.75	3	
		KVAPIQR	955.61	1	
		ILFFNDK	1040.59	1	

**145**	**O75410**	**Transforming acidic coiled-coil-containing protein 1**			
		LGSTLTPK	960.58	1	
		KNEEALK	975.56	1	
		ANEEIAQVR	1029.53	----	
		ICDELIAK	1048.58	1	
**146**	**Q01995**	**Transgelin**			
		KAQEHK	1028.61	2	
		LGFQVWLK	1134.68	1	
		QMEQVAQFLK	1348.71	1	Gln->pyro-Glu@N-term
		AAEDYGVIK	1253.70	2	
		GSKPVK	903.57	2	
		KYDEELEER	1354.66	1	
		KYDEELEER	1498.76	2	
		LGFQVWLK	1134.68	1	
		LGFQVWLK	1278.78	2	
		QMEQVAQFLK	1356.67	1	Dethiomethyl(M)@2, Cation:K(E)@3 Deamidated(Q)@4
**147**	**Q9UJA5**	**tRNA (adenine(58)-N(1))-methyltransferase non-catalytic subunit TRM6**			
		ADTSLK	778.44	1	
		QEEQRK	961.52	1	
		AATACFGFPK	1156.59	1	
		RHLEAAALLSER	1365.76	----	
**148**	**Q9NYL9**	**Tropomodulin-3**			
		GKIFIPK	1090.72	2	
		NNDLVRK	1002.58	1	
		ALETNTHVK	1156.64		
		QKPVQTFTEEK	1622.89	2	
**149**	**P06753**	**Tropomyosin alpha-3-chain**			
		AEFAER	836.45	1	
		YEEEIK	954.49	1	
		HIAEEADRK	1212.64	1	
		YEEEIKILTDK	1668.92	2	
**150**	**P07951**	**Tropomyosin beta chain**			
		AMKDEEK	1138.60	2	
		EEKLK	934.58	2	
		IQLVEEELDR	1243.65	----	
		KYEEVAR	1038.57	1	
		AEFAERSVAK	1251.68	1	
		LLEEKLK	1016.64	1	
		LVILEGELER	1170.67	----	
**151**	**P49815**	**Tuberin**			
		DSGLKEK	1064.62	2	
		ALFFKVIK	1253.82	2	
		ATASNAEK	935.49	1	
		VERDALK	974.57	1	
		SNPTDIYPSK	1265.65	1	
**152**	**P07437**	**Tubulin beta chain**			
		GRMSMK	853.45	1	
		ISEQFTAMFR	1229.59	----	
		TAVCDIPPRGLK	1413.80	1	
		KLAVNMVPFPR	1415.83	1	
**153**	**P78324**	**Tyrosine-protein phosphatase non-receptor type substrate 1**			
		SGAGTELSVR	976.51	----	
		AENQVNVTCQVR	1361.64	----	Deamidated(N)@6
		LHEPEKNAR	1238.66	1	Deamidated(N)@7
		VPPTLEVTQQPV	1463.82	----	
		TETASTVTENK	1324.67	1	
		LHEPEK	896.49	1	
**154**	**Q9NPG3**	**Ubinuclein-1**			
		AAEGESRQK	1120.57	1	Deamidated(Q)@8
		DKEKK	1079.68	3	
		EEDEEK	922.41	1	
		GGRRIMGPRK	1287.75	1	Oxidation(M)@6
		KDDTYDK	1172.60	2	

		KEKK	964.65	3	
		KKKSPKK	1275.88	3	
		KYSGALSVK	1240.75	2	
		VFHAGTQQQK	1287.69	1	
		YGGKKRRK	1280.81	2	
**155**	**Q14139**	**Ubiquitin conjugation factor E4 A**			
		SSPQEIK	932.51	1	
		AAIQKEQLK	1172.71	1	
		EERKIK	1090.68	2	
		EYIQPK	921.52	1	
		IYQMLK	939.55	1	
		NPSRSSPQEIK	1386.75	1	
		SSPQEIK	949.49	1	Oxidation(P)@3, Deamidated(Q)@4
**156**	**Q9Y6A4**	**Cilia- and flagella-associated protein 20**			
		AYGTNYIETLR	1300.65	----	
		KVRNGHIK	1240.77	2	Deamidated(N)@4
		LYLPVQNK	1118.67	1	
		LYLPVQNKAK	1717.10	2	
		LPFLVMIIKNLK	1573.01	1	
**157**	**Q15849**	**Urea transporter 2**			
		RASIITK	932.60	1	
		AVGYLTGDMK	1198.62	1	
		ECGEGLKDK	1122.56	1	
		IYYLSQERNR	1341.69	----	
**158**	**Q8N6Y0**	**Usher syndrome type-1C protein-binding protein 1**			
		GSIEKLK	918.57	1	
		ARRSQSAELNR	1287.69	----	
		GSIEKLK	918.57	----	
		IEKLK	918.62	2	
		IMEAQMEQLR	1249.59	----	Deamidated(Q)@8
		MSARATRPR	1189.67	1	
		REKRGLELR	1300.79	1	
		RSQSAELNR	1060.55	----	
**159**	**P62955**	**Voltage-dependent calcium channel gamma 7 subunit**			
		VCFFAGREK	1200.63	1	
		EGAGVMSVYLFTK	1545.81	1	
		EGAGVMSVYLFTKR	1701.91	1	
**160**	**P21281**	**V-type proton ATPase subunit B, brain isoform**			
		SGQVLEVSGSK	1090.57	----	
		TPVSEDMLGR	1120.53		Oxidation(M)@7
		SAIGEGMTRK	1193.64	1	
		VFNGSGKPIDR	1303.73	1	
		QAGLVKK	887.58	1	
**161**	**Q9UJW8**	**Zinc finger protein 180**			
		SGLNSSLFSSPVIPIR	1673.92	----	
		KQQEK	949.56	2	Deamidated(Q)@3
		IPFEESQYK	1284.66	1	
		HQATHTEEK	1224.61	1	
		IHGGGKTFDFK	1350.72	1	
**162**	**Q7Z3V5**	**Zinc finger protein 571**			
		GSQLTEHQR	1056.51	----	Deamidated(Q)@3
		GSQLTEHQR	1055.52	----	
		CKECGK	811.39	1	
		ECGKTFVR	1083.57	1	
		ECGKAFICGK	1199.60	1	
**163**	**Q9H582**	**Zinc finger protein 644**			
		RFVQK	821.51	1	
		DSVVGSSK	922.49	1	
		RTGTGTPVK	1060.62	1	
		YFHQAAKEK	1265.67	1	
		NQSLTLIELLK	1415.86	1	
**164**	**Q15776**	**Zinc finger protein with KRAB and SCAN domains 8**			
		SGLIEHQR	939.50	----	
		RSHTGEKP	1055.57	1	
		SFAQSSGLVR	1052.54		Deamidated(Q)@4
		SENRELASK	1147.63	1	

		HQRIHSGEK	1235.67	1	

Table 3S: Ipa data set.

[A] **ANALYSIS SETTING**
Reference set: Ingenuity Knowledge Base (Genes Only)<br>Relationship to include: Direct and Indirect<br>Includes Endogenous Chemicals<br>Optional Analyses: My Pathways My List<br>Consider only molecules and/or relationships where<br>(species = Human) AND<br>(confidence = Experimentally Observed)

[B] **Top Canonical Pathways:**

**Name**	**p-value**	**Ratio**
Epithelial Adherens Junction Signaling	3,09E-04	7/143 (0,049)
Role of Wnt/GSK-3β Signaling in the Pathogenesis of influenza	5,02E-04	5/73 (0,068)
P13K/AKT Signaling	7,81E-04	6/121 (0,05)
Calcium Signaling	8,69E-04	7/170 (0,041)
Cellular Effects of Sildenafil (Viagra)	8,88E-04	6/124 (0,048)

[C] **Top Upstream Regulator:**

**Name**	**p-value**
MYOCD	6,93 E-05
LDL	2,8E-04
miR-145-5p	1,72E-03
HAND2	2,91E-03
TBX5	2,91E-03

[D] **Top Disease and BioFunction**

**Name**	**p-value**	**Molecules**
Cancer	2,81E-07 – 1,79E-02	134
Gastrointestinal Disease	2,81E-07 – 1,79E-02	84
Organismal Injury and Abnormalities	7,10E-06 - 1,79E-02	93
Reproductive system disease	7,93E-06 - 1,79E-02	79
Renal and Urological Disease	1,39E-05 – 1,37E-02	17

[E] **Molecular and Cellular Functions**

**Name**	**p-value**	**molecules**
Cellular movement	9,50E-06 – 8,98E-03	35
Cellular assembly and organization	1,25E-04 – 1,79E-02	24
Cellular development	1,33E-04 – 1,79E-02	32
Cellular growth and proliferation	1,33E-04 – 1,79E-02	28
Gene expression	1,48E-04 – 1,79E-02	31

[F] **Top Networks**

**Associated Network Functions**	**Score**
Cell Death and Survival, Cancer, Gastrointestinal disease	78

Cancer , Cellular growth and proliferation, Organism Injury and abnormalities,	30
Cardiovascular System development and function, cellular movement, cardiac infarction	30
Gene expression Cell Death and Survival, Cancer	18
Cell Morphology, Cellular Development, Digestive System development and function	2

[G] Top Molecules

Fold Change up-regulated

**Molecules**	**Exp Value**
MAFA	9.408
HIPK3	9,073
BCAR3	7,210
MYH13	6,928
GPR144	6,807
DPYS	6,304
DMXL1	6,136
RBMS1	6,070
ARAP1	5,780
TACC1	5,550

Fold Change down-regulated

**Molecules**	**Exp. value**
KIN	-1,988
GOLGB1	-1,988
TET1	-1,493
TPM2	-1,484
KCNH7	-1,484
C16orf80	-1,077
INSIG1	-1,020
TAGLN	-1,002
